# Spatiotemporal Brain Dynamics During Cyclic Seizures of Super‐Refractory Status Epilepticus

**DOI:** 10.1111/ene.70428

**Published:** 2025-11-26

**Authors:** Alexandre Ledos, Gaspard Martet, Maeva Le Goïc, Sophie Demeret, Mario Chavez, Virginie Lambrecq

**Affiliations:** ^1^ Department of Anesthesiology and Critical Care Pontchaillou Hospital Rennes France; ^2^ Paris Brain Institute, CNRS‐UMR7225, Inserm U1127 Sorbonne University UM75 Paris France; ^3^ Department of Neurophysiology, DMU Neurosciences La Pitié‐Salpêtrière Hospital Paris France; ^4^ Paris Brain Institute, Data Analysis Core, INSERM, CNRS Sorbonne Université, Pitié‐Salpêtrière Hospital Paris France; ^5^ Neuro Intensive Care Unit, DMU Neurosciences La Pitié‐Salpêtrière Hospital Paris France

**Keywords:** critical care, electroencephalography, electroencephalography phase synchronization, seizures, status epilepticus

## Abstract

**Background:**

Cyclic seizures (CS) are defined by the periodic recurrence of epileptic activity and may offer critical insights into the mechanisms of seizure termination. Previous studies have reported increased network synchronization during the final stages of seizures. The objective of this study was to investigate hypersynchronization at the termination of cyclic seizures and to characterize its spatial distribution.

**Material and Methods:**

To quantify brain synchronization, we calculated the imaginary coherence in a low‐frequency band (LF‐iCOH) at different ictal phases. Synchronization was calculated at both scalp regions and electrode levels. A total of 10 patients were selected, and 1230 seizures from 468 h of EEG monitoring were analyzed.

**Results:**

Eight patients exhibited a significant increase in LF‐iCOH at seizure termination in both regional and electrode‐level analyses, with varying topographies. Hypersynchronization at the end of seizures was predominantly observed in the anterior and centrotemporal regions compared to the posterior regions.

**Discussion:**

Terminal hypersynchronization was observed in all patients with focal to bilateral seizures and one patient with focal seizures, and was not observed in patients with localized focal seizures. The diverse topographies of terminal hypersynchronization observed suggest that this process is not related to any specific scalp area or seizure onset zone. These results suggest a common mechanism of seizure termination in the continuum of single seizures to the most severe form of SE.

## 1 Background

Status epilepticus (SE) is a life‐threatening neurological condition resulting from either failure of the mechanisms responsible for seizure termination or from the initiation of mechanisms which lead to abnormally prolonged seizures [1]. Approximately 10%–30% of SE cases progress to refractory status epilepticus (RSE) [2]. Super‐refractory status epilepticus (SR‐SE), defined as the persistence of convulsions or electrical seizures despite a 24‐h therapeutic coma, is responsible for increased in‐hospital mortality [3] and neuronal injury [4]. Continuous electroencephalography (cEEG) with scalp electrodes is a major tool for monitoring patients with RSE and SR‐SE to detect seizures and monitor the effects of antiseizure medications. It is well established that many seizures in comatose patients in the ICU are non‐convulsive [5]. The growing use of cEEG in SE patients admitted to the ICU has led to the description of a particular pattern of periodic recurrence of seizures, known as “cyclic seizures”, characterized by the periodic recurrence of electrographic seizures at regular intervals, typically greater than three seizures per hour for at least 1 h [6].

Cyclic seizures (CS) were initially described in 13 critically ill patients [6], characterized by frequent recurrence of electrographic seizures often organized in clusters of multiple discharges. Subsequent studies have linked CS with acute or progressive brain injury, typically originating focally, and with a potentially poorer prognosis [7]. However, a more recent study suggested that CS may have a more favorable outcome in patients under 75 years of age [8]. Therefore, the prognostic significance of CS remains uncertain and requires further investigation.

CS could be key to understanding seizure termination, as they may reflect the incapacity of seizures to become continuous as well as to be able to stop. However, the mechanism of seizure termination remains poorly understood [9]. The pathophysiology of SE is complex and involves multiple mechanisms at both cellular and network levels [10]. Coordinated neuronal interactions are essential for the normal functioning of the brain [11] and altered synchronization plays a major role in the pathophysiology of epilepsy [12]. High levels of synchronization have been observed prior to the termination of seizures across different in vitro and in vivo models [13, 14], as well as in human seizures [15, 16, 17]. Notably, a study examining the synchronization dynamics in non‐refractory status epilepticus, found that global synchronization increased before the end of the seizures [16]. This hypersynchronization could reflect the self‐regulatory mechanisms of seizure termination: the end of focal seizures is characterized by large amplitude activity synchronized in time and in space [18]. However, there are no data in the literature concerning the spatial distribution of this increasing synchronization at seizure termination.

The primary objective of the present study was to investigate hypersynchronization at the termination of seizure in cyclic seizures and to map the localization of this terminal hypersynchronization. Using the CS model of recurrent stereotyped seizures, we aimed to shed light on the spatial dynamics of seizure terminating, potentially offering new insights to underlying mechanisms that regulate seizure cessation.

## 2 Methods

### 2.1 Patient's Selection

Patients were retrospectively selected from the MONITOBA database, which contains data from individuals admitted between 2015 and 2023 to the neurological intensive care unit at La Pitié‐Salpêtrière Hospital (Paris) and undergoing continuous EEG monitoring. A total of 308 patients were monitored for SE. Among the patients admitted and monitored for SR‐SE, 20 were identified as presenting cyclic seizures. Patients were included regardless of the etiology of status epilepticus. The following clinical data were collected: age, sex, history of epilepsy, etiology of seizure, length of ICU stay, ICU mortality, and number of hypnotic and antiseizure medications used during cyclic seizures. This study was reviewed and approved by CHU Pitié‐Salpêtrière ethic local commissions. The database is kept by the hospital (register number: 20240327134527), in accordance with CNIL recommendations.

### 2.2 EEG Recordings

EEG recordings were carried out in neuro‐ICU 24 h a day with a standard 8‐electrode setup, over periods of more than 48 h. Continuous monitoring was provided by a standard clinical EEG‐video system (Micromed, software: System Plus Evolution or Compumedics, software: Profusion EEG), using 8 Ag/AgCl cup electrodes placed on the scalp according to the international 10–20 system (left hemisphere: Fp1‐C3‐T3‐O1 and right hemisphere: Fp2‐C4‐T4‐O2). EEG signals were sampled at 256 Hz, A/D converted at 12‐bit resolution, bandpass filtered between 0.5–70 Hz, with a notch filter at 50 Hz.

Electrographic seizures were defined according to the standardized critical care terminology of the ANCS guidelines [19]. Seizures were selected for analysis using the following criteria: presence of cyclic seizures, limited incidence of artifacts, number of consecutive electrographic seizures > 50, duration of seizure more than 20 s and stereotyped seizures presenting the same seizure onset zone (SOZ) and propagation patterns. Patients were excluded if more than 30% of seizures were artifacted with movements or if seizures were multifocal with multiple SOZ.

### 2.3 Data Preprocessing

**FIGURE 1:**
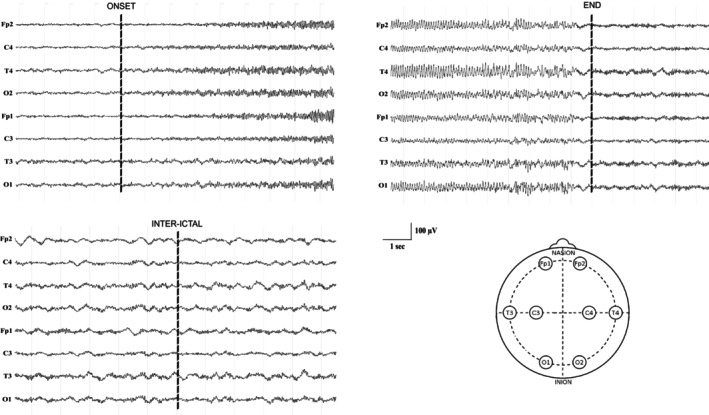
Preprocessing of raw EEG. Recordings of 8 monopolar channels showing the placement of markers to define ictal phases of interest. Seizure onset marker was placed at the appearance of the epileptic activity. Seizure termination marker was placed at the end of the rhythmic discharge. Inter‐ictal markers were placed between seizures.

**FIGURE 2:**
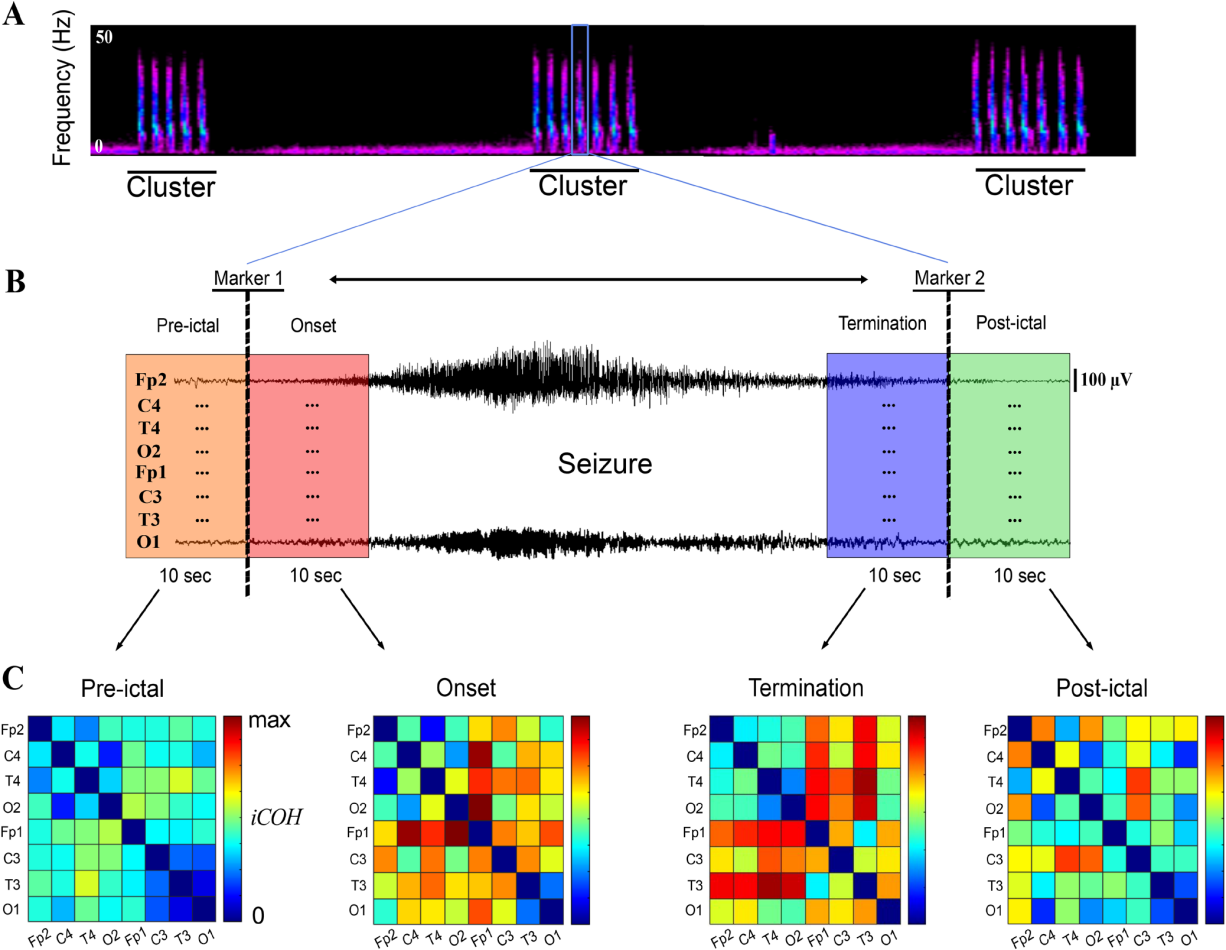
Preprocessing and estimation of synchronization (A) Spectrogram visualization of three clusters of cyclic seizures. (B) One seizure recorded with eight electrodes, displayed with a monopolar montage. Four phases of 10 s duration were defined by positioning the seizure onset and termination markers (pre‐ictal, onset, termination, and post‐ictal). (C) Connectivity matrices were obtained by calculating the low frequency imaginary coherence (LF iCOH) between each combination of electrodes in the [1–8] Hz band, for each ictal phase.

Raw EEG recordings were visually analyzed and annotated using the open‐source software EDFbrowser (version 1.98). Each selected cyclic seizure was annotated using the following markers: seizure onset (defined as the appearance of the epileptic discharge) and seizure termination (defined as the cessation of the epileptic discharge) (Figure 1). These annotations were verified by expert neurophysiologists. Time‐frequency tools were performed on each EEG file via the short‐time Fourier Transform to identify seizures with a cyclic pattern (Figure 2A).

Then, markers were placed in the inter‐ictal period according to the following criteria: seizure‐free period, at least 5 min apart from a seizure, and artifacts limited. The locations of seizure onset and termination, seizure type, and mean seizure duration were recorded for each patient.

### 2.4 Synchronization

Volume conduction is defined as the diffusion of electric or magnetic fields through biological tissues. Thus, the activity of a single generator within the brain can typically be observed in many EEG scalp channels. The relation between channels measured by linear coherence is rather a trivial volume conduction artifact than a reflection of a true brain interaction. One approach to attenuate artifacts due to volume conduction is to use methods blind to instantaneous connectivity, like the imaginary coherence [20].

Coherence is a measure of the linear relationship between two EEG signals at a specific frequency, obtained from the Fourier transforms of time series from two EEG channels. Coherence is a complex number with both real and imaginary parts. By excluding the real part and retaining only the imaginary component, we obtained the imaginary coherence (iCOH). This measure excludes the instantaneous interactions that are potentially spurious due to volume conduction. iCOH is therefore only sensitive to interactions of two processes that are time‐lagged to each other, which provide a more robust estimator of functional connectivity [20].

iCOH was calculated using MATLAB (MathWorks Inc., version R2022a), over 10‐s windows preceding each marker and over 10‐s windows following each marker, between each pair of monopolar EEG signals (i.e., 28 combinations) in two frequency bands: delta (1–4 Hz) and theta (4–8 Hz). The iCOH in the low frequency (LF) band (1–8 Hz) was obtained by calculating the mean of iCOH from the delta band and the theta band. The measurement obtained was an iCOH value that ranged from 0 (no correlation) to 1 (perfect synchronization) in the LF band. The choice of this specific frequency band was justified by a preliminary analysis that showed a minimal rate of artifacts in the LF band.

### 2.5 Regional and Electrodes Analysis

**FIGURE 3:**
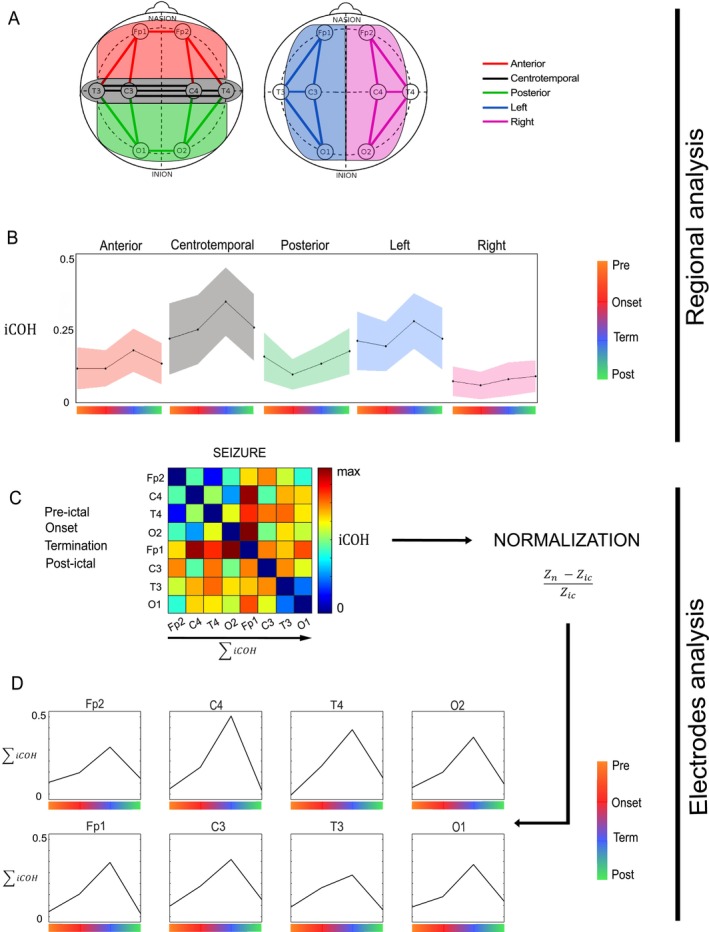
Regional and electrodes analyses (A) Regional connectivity of brain areas was estimated by calculating the average of iCOH from five pairs of electrodes. (B) Evolution of regional LF ICOH averaging for all seizures (±SD). (C) Connectivity matrix obtained by calculating the LF iCOH between each combination of electrodes in the [1–8] Hz band. (D) Changes in total synchrony in each electrode at the different ictal phases. LF iCOH sum was calculated for each electrode and normalized to the average value of all interictal periods.

iCOH was estimated for different scalp regions to obtain anatomical divisions. Each synchrony region was calculated from iCOH values of five pairs of electrodes per area: anterior, centrotemporal, posterior, left, and right (Figure 3A). Then, we obtained a local average of iCOHs for each region in anteroposterior segmentation (anterior, centrotemporal and posterior) and an average iCOH for each hemisphere (left and right). This allowed us to study changes, in regional synchronization during the different ictal phases (Figure 3B).

For each seizure phase of interest (pre‐ictal, onset, termination, post‐ictal) (Figure 2B), a connectivity matrix of dimensions 8 × 8 was obtained (Figure 2C), representing the iCOH value in the LF band between all pairs of electrodes. A connectivity matrix for the inter‐ictal period was also obtained from the average of all the inter‐ictal windows. Each entry of the connectivity (iCOH) matrix was adjusted over the inter‐ictal period using the following formula:

$$\frac{Z_{n}-Z_{\mathrm{ic}}}{Z_{\mathrm{ic}}}$$

where *Z*_*n*_ is the initial iCOH value and *Z*_*ic*_ denotes the average over all interictal periods.

From each iCOH connectivity matrix, the calculation of the sum of each line provides the total coherence for each electrode at a given ictal phase (Figure 3D). The iCOH sum thus represents the total connectivity of each electrode normalized to the baseline connectivity level during the inter‐ictal phase.

### 2.6 Statistical Analyses

Statistical analyses were conducted using R version 4.2.2. Group comparisons were performed using linear mixed‐effect models (LMM) [21] to study the seizure phase (pre‐ictal, onset, termination, post‐ictal) and localization effects with their interaction term on the iCOH variations. The localization of EEG signals has been studied either for all electrodes or in terms of five regions defined by the corresponding average iCOH value (anterior, central, posterior, left and right). Several models have been adjusted for each patient and for the entire group of patients, considering seizure numbers and subject identifiers as random intercept effects. All LMMs were fitted using restricted maximum‐likelihood estimation (REML). Significance of the main and interaction effects was assessed using Type II Wald chi‐square tests with Kenward‐Roger degrees of freedom using the Anova function. Natural logarithm or square‐root transformations were applied when appropriate to improve the fit of the model. Post hoc pairwise comparisons were then conducted between the ictal periods at each localization with Dunnett's correction for multiple comparisons against the termination phase. Model assumptions were checked visually based on the residuals' plots. All the tests were two‐sided and the level of statistical significance was set at *p* or adjusted *p* < 0.05.

## 3 Results

### 3.1 Clinical Results

Of the 20 patients with cyclic seizures, 10 met the selection criteria and were selected for cyclic seizure analysis. Ten patients were excluded due to an insufficient number of cyclic seizures (< 50), a significant presence of artifacts or multifocal seizures. None of the patients had a history of epileptic seizures. Regarding the causes of SR‐SE, six patients had cryptogenic encephalitis (probable seronegative autoimmune encephalitis), two had confirmed autoimmune encephalitis, one had Posterior Reversible Encephalopathy Syndrome, and one had viral encephalitis. They were critical care patients with severe illness, requiring prolonged stays (60 days on average) and contributing to 3 ICU deaths in the sample. All patients were sedated and ventilated during the recording of the analyzed cyclic seizures. On average, each patient was administered four antiseizure medications.

We reviewed and annotated 468 h of cEEG recordings to analyze 1230 cyclic seizures from 10 patients (62–306 seizures per patient). The majority of patients had focal to bilateral electrographic seizures (previously known as secondarily generalized seizures) (7/10) and three patients had focal seizures without bilateralization. The average seizure duration was 118 s (46–182 s). Five patients presented with cyclic seizures organized in clusters. All seizures were nonconvulsive. Detailed clinical and EEG information is given in Table 1.

**TABLE 1:** Clinical information and electrophysiological features.

Patient	Age, gender	Duration of ICU stay (days)	ICU‐death	Status epilepticus etiology	MRI findings	Seizure type	Clustered seizures	Analyzed seizures	Mean duration of seizures (seconds ± SD)	Recording duration (hours)	Administrated hypnotics
01	20, M	65	No	AE	No HS	FB	Yes	258	97 (±30)	152	PRO, MDZ
02	35, M	93	No	CE	Bitemporal FLAIR HS	FB	Yes	62	126 (±36)	45	PRO, MDZ
03	32, F	64	No	CE	Cortical & pulvinar diffusion HS	FB	Yes	106	70 (±16)	24	PRO, MDZ, KET, ISO
04	39, M	46	Yes	CE	Limbic and temporal FLAIR HS	FB	Yes	72	119 (±16)	21	MDZ, KET
05	22, M	67	No	CE	Bilateral hippocampi diffusion & FLAIR HS	FB	Yes	68	89 (±49)	71	PRO, MDZ, KET
06	20, M	126	No	CE	Lenticular & caudate nuclei T1 HS/external capsule FLAIR HS	FB	No	62	163 (±13)	10	PRO, MDZ
07	26, M	38	No	CE	Left mesial temporal FLAIR HS	FB	No	84	145 (±18)	57	PRO, MDZ
08	51, M	36	Yes	VE	Bi‐parietal & left mesial temporal FLAIR HS	F	No	306	46 (±15)	29	MDZ
09	18, M	17	No	AE	Left fronto‐parietal cortex, right opercular & left hippocampus FLAIR HS	F	No	120	144 (±59)	39	PRO
10	59, M	48	Yes	PRES	Right posterior FLAIR HS	F	No	92	182 (±52)	20	PRO

Abbreviations: AE, autoimmune encephalitis; CE, cryptogenic encephalitis; F, focal seizures; FB, focal to bilateral seizures; FLAIR, Fluid attenuated inversion recovery; HS, hypersignal; ISO, isoflurane; KET, ketamine; MDZ, midazolam; PRES, Posterior Reversible Encephalopathy Syndrome; PRO, propofol; VE, viral encephalitis.

### 3.2 Regional Analysis

Eight patients showed significant increases in LF iCOH at the end of seizures, particularly in patients with focal to bilateral seizures.

The LMM analysis was first applied globally on all seizures with all patients combined. Type II Wald chi‐square tests revealed an interaction effect of the ictal phases (pre‐ictal, onset, termination and post‐ictal) and the scalp regions (anterior, central, posterior, left and right) (*p* < 0.001) on iCOH variations for all seizures. The LMM was then applied separately to seizures of each patient (Table 2).

**TABLE 2:** Analysis of deviance table (Type II Wald Chi‐Squared tests) of linear mixed‐effects models.

Patient	Regions analysis			Electrodes analysis		
	Variable	*χ* ^2^	*p*	Variable	*χ* ^2^	*p*
All patients	States	882.26	**< 0.0001**	States	739.64	**< 0.0001**
	Regions	3189.45	**< 0.0001**	Electrodes	107.30	**< 0.0001**
	States*Regions	139.54	**< 0.0001**	States*Electrodes	69.01	**< 0.0001**
Patient 1	States	745.19	**< 0.0001**	States	1398.98	**< 0.0001**
	Regions	856.55	**< 0.0001**	Electrodes	30.78	**0.0001**
	States*Regions	43.07	**< 0.0001**	States*Electrodes	63.13	**< 0.0001**
Patient 2	States	248.97	**< 0.0001**	States	565.83	**< 0.0001**
	Regions	223.30	**< 0.0001**	Electrodes	20.98	**0.0038**
	States*Regions	8.90	0.71	States*Electrodes	25.62	0.22
Patient 3	States	38.90	**< 0.0001**	States	296.69	**< 0.0001**
	Regions	163.25	**< 0.0001**	Electrodes	59.53	**< 0.0001**
	States*Regions	47.36	**< 0.0001**	States*Electrodes	137.43	**< 0.0001**
Patient 4	States	383.13	**< 0.0001**	States	686.65	**< 0.0001**
	Regions	251.01	**< 0.0001**	Electrodes	57.55	**< 0.0001**
	States*Regions	44.79	**< 0.0001**	States*Electrodes	72.67	**< 0.0001**
Patient 5	States	72.53	**< 0.0001**	States	127.05	**< 0.0001**
	Regions	120.65	**< 0.0001**	Electrodes	19.05	**0.008**
	States*Regions	10.54	0.57	States*Electrodes	13.86	0.88
Patient 6	States	104.04	**< 0.0001**	States	242.04	**< 0.0001**
	Regions	472.07	**< 0.0001**	Electrodes	57.93	**< 0.0001**
	States*Regions	73.69	**< 0.0001**	States*Electrodes	135.14	**< 0.0001**
Patient 7	States	219.73	**< 0.0001**	States	549.52	**< 0.0001**
	Regions	126.47	**< 0.0001**	Electrodes	67.23	**< 0.0001**
	States*Regions	139.35	**< 0.0001**	States*Electrodes	176.28	**< 0.0001**
Patient 8	States	515.73	**< 0.0001**	States	797.65	**< 0.0001**
	Regions	5405.45	**< 0.0001**	Electrodes	460.36	**< 0.0001**
	States*Regions	416.35	**< 0.0001**	States*Electrodes	411.83	**< 0.0001**
Patient 9	States	44.49	**< 0.0001**	States	21.38	**0.0001**
	Regions	1868.90	**< 0.0001**	Electrodes	8.18	0.32
	States*Regions	5.30	0.95	States*Electrodes	10.52	0.97
Patient 10	States	30.86	**< 0.0001**	States	5.40	0.15
	Regions	1586.21	**< 0.0001**	Electrodes	17.38	**0.015**
	States*Regions	46.19	**< 0.0001**	States*Electrodes	25.25	0.24

*Note:* Linear mixed‐effects models were performed to study the seizure state (pre‐ictal, onset, termination, post‐ictal) and localization effects with their interaction term on the iCOH variations. The localization of EEG signals has been studied either for five regions (anterior, central, posterior, left, and right) and for all electrodes (Fp1, Fp2, C4, T4, O2, C3, T3, O1) defined by the corresponding average iCOH value. *p* < 0.05 was considered significant. Results with *p*‐values below the significance threshold are displayed in bold.

Multiple comparisons using Dunnett's method were performed independently for each patient. Patients #1 to #8 showed a significant increase in terminal iCOH in at least one brain region. In these patients, the right hemisphere was more involved (6/8 patients) than the left (3/8 patients). The anterior and centrotemporal regions were involved in 5/8 patients each. The posterior region was not affected by this terminal hypersynchronization. Seven of these eight patients experienced focal to bilateral seizures. Terminal hypersynchronization was not correlated with the localization of the SOZ.

In patients with focal seizures without bilateralization (patients #8–10), statistical analysis showed a significant increase in iCOH at seizure termination in only one subject (patient #8). This patient had seizures starting in the left posterior cortex and spreading to the entire left hemisphere, unlike the other two patients, with no propagation observed on the EEG recordings. Results of the regional analysis compared to the visual interpretation of EEG are presented in Table 3.

**TABLE 3:** Description of seizures and results of regional analyses.

Patient	EEG visual analysis		Terminal hypersynchronization at regional analysis	
	Onset (SOZ)	Termination	Scalp regions	Hemispheres
01	Anterior	Diffuse	Anterior, centrotemporal	Left, right
02	Right centrotemporal	Diffuse	Anterior, centrotemporal	Left, right
03	Right posterior	Diffuse	Centrotemporal	—
04	Anterior	Anterior	Anterior, centrotemporal	Right
05	Right hemisphere	Diffuse (unless Fp1/C3)	—	Right
06	Left centrotemporal	Temporo‐posterior	Anterior	Right
07	Anterior	Right hemisphere	—	Right
08	Left temporo‐posterior	Left hemisphere	Anterior, centrotemporal	Left
09	Left posterior	Left posterior	—	—
10	Right posterior	Right posterior	—	—

*Note:* EEG visual analysis: Localization of seizure onset and termination in scalp regions based on visual raw EEG analysis. Terminal hypersynchronization at regional analysis: Localization of increased estimated synchronization in scalp regions and hemispheres at the end of seizures.

Abbreviations: F, focal seizures; FB, focal to bilateral seizures; SOZ, seizure onset zone.

### 3.3 Electrode Analysis

Electrode analyses showed similar results to those obtained from regional analyses. The LMM analysis was applied globally on all seizures with all patients combined to identify possible interaction effects between ictal phases, electrodes and iCOH variations. Statistical tests revealed a significant effect of the seizure phase (*p* < 0.001) and electrode localization (*p* < 0.001) on iCOH variations. The overall LMM also showed an interaction effect between seizure phases and electrodes (*p* < 0.001) on iCOH variations for all seizures. The LMM was then applied separately to seizures of each patient (Table 2).

**FIGURE 4:**
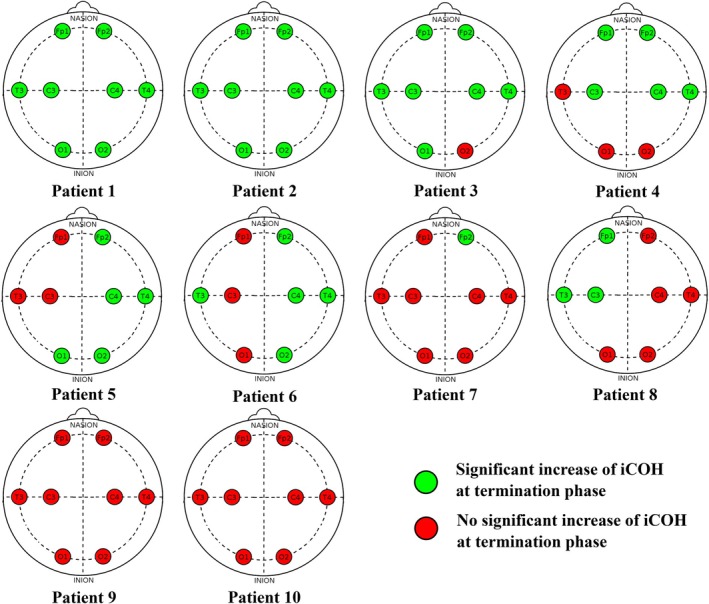
Electrodes analysis for each patient. Significant increase (or not) of LF iCOH adjusted sum at seizure end.

Multiple comparisons using Dunnett's method were then performed independently for each patient. Statistical analysis showed a significant increase of iCOH at seizure termination on at least one electrode in eight patients (Figure 4). Detailed statistical analysis is shown in Appendix S1.

For each of these eight patients (patients #1–8), at least one of the two frontal electrodes was consistently affected by terminal hypersynchronization. Two patients showed a significant increase in iCOH preceding the end of seizures on all electrodes (patients 1 and 2), with a significant bilateral increase in the anterior and central regions on the regional analysis. Four patients showed less diffuse terminal synchronization, with significantly higher iCOH at the end of the seizure on more than four electrodes (patients #36), and two patients showed more localized hypersynchronization on one and three electrodes (patients #7 and 8, respectively).

The two patients for whom electrode analysis did not identify terminal hypersynchronization were also those without a significant increase in terminal iCOH on regional analysis (patients #9 and 10).

When analyzing the distribution of terminal hypersynchronization based on electrode localization, the frontopolar (Fp) electrodes most frequently exhibited significant changes (12/20 electrodes), followed by the central (C) electrodes (11/20) and the temporal (T) electrodes (10/20). In contrast, the occipital (O) electrodes demonstrated the lowest occurrence of a significant increase in iCOH at seizure termination (8/20).

## 4 Discussion

In eight of the 10 patients, we found a significant increase in synchronization at the end of cyclic seizures. This terminal seizure hypersynchronization exhibited various scalp topographies, at brain regions as well as electrode levels. To the best of our knowledge, this is the first study to evaluate the spatial distribution of terminal seizure hypersynchronization in super‐refractory status epilepticus.

In humans, several studies have reported hypersynchronization at the final stage of focal seizures [17] and non‐refractory SE [16] using different methods of estimating functional connectivity. Liu and Zhang [17] used the variations of phase synchronization while Schindler et al. [16] used the relative changes of the eigenvalue spectrum of the equal‐time correlation matrix. Our results obtained with LF iCOH in SR‐SE patients align with these previous findings, supporting the hypothesis of a common mechanism involved in the continuum from single seizure to one of the most severe forms of SE. Despite the small number of subjects, our results were consolidated by analyzing a large number of seizures using rigorous statistical methods.

Results of the regional analysis indicate that anterior and centrotemporal regions were most concerned by terminal hypersynchronization while the posterior region showed no significant increase in synchronization at the end of seizures. The electrode analysis found various topographies of significant increased synchronization at the end of seizure: whole cortical involvement, widely distributed and localized terminal hypersynchronization. Patients with clustered seizures (#1–5) had whole cortex or widely distributed terminal hypersynchronization.

Terminal hypersynchronization was found in seven patients with focal‐to‐bilateral seizures and in one patient with focal seizures propagating to the left whole hemisphere. This pattern was not found in two patients presenting focal seizures that did not bilateralize (patients #9 and 10); these seizures were characterized by a posterior onset without spatial propagation of the epileptic activities. It is known that parietal and occipital seizures are challenging to study due to their rapid propagation; furthermore, posterior cortex seizures are the least common form of focal epilepsy [22]. Given the limited 8‐electrode montage used in this study, the spatial resolution for detecting very localized increases in synchronization was restricted. However, terminal hypersynchronization might not be implied in localized seizures, as most of the previously mentioned studies included patients with propagating seizures [16, 17].

It has been proposed that the ictal increase of synchronized neuronal activity may be an emergent self‐regulatory mechanism for seizure termination. Several studies have shown that seizures terminate over large brain areas with the recruitment of large neuronal populations in the final phase of seizures at the network level [13, 14, 23]. In our study, six patients presented a wide distribution (≥ 5 electrodes) of increased synchronization at seizure end. One possible explanation is that the increasing involvement of inhibitory interneurons could cause recovery of potent synchronized inhibition and seizure termination [12]. In focal seizures, a transition from tonic firing to rhythmic bursting is constantly observed with typical evolution from high to lower frequencies [18]. This transition to large and slow amplitude activity synchronized in time and in space at the end of seizure could be related to synchronized inhibition by interneurons [9]. In a recent review [24] investigating the role of GABAergic interneurons during seizures, low frequency (1 Hz) optogenetic stimulation was found to be anti‐epileptic while high‐frequency stimulation (20 Hz) was found to be pro‐epileptic. Therefore, an increase in EEG synchronization seems to be crucial for seizure termination and it has even been experimented with as an antiseizure mechanism: low‐frequency electric stimulation of the seizure onset zone was tested in 10 patients and significant changes in EEG synchronization were observed in 74% of stimulations [25].

The limitations of our analysis are primarily related to the use of surface EEG, which is highly sensitive to movement artifacts. However, most patients were sedated during EEG recordings, which helped minimize this issue. Moreover, intravenous hypnotics alter brain synchronization at multiple levels. Typically, high doses of sedative agents result in the attenuation of fast rhythms and an increase in slow‐frequency activity on scalp EEG. Although the effects of general anesthetics on functional connectivity vary depending on the agent, dosage, and neural network examined [26], the existing literature on EEG synchronization changes during seizures under sedation remains limited. Based on these considerations, we hypothesize that the use of deep sedation may have contributed to the enhancement of low‐frequency functional connectivity observed in our study. Furthermore, the critical care environment provides high rates of extracerebral signals from various devices (e.g., ventilators, scopes…). To address this, the low‐frequency band was chosen because of its minimal artifact rate, and all seizures were carefully reviewed to avoid artifact contamination. The montage used in the neuro‐ICU for EEG‐monitoring was simplified with eight electrodes which, while limited, provide a large quantity of data with long‐term recordings. Furthermore, the use of a reduced electrode montage may underestimate localized connectivity patterns in deeper or mesial regions. Volume conduction is a well‐known problem in the EEG and signal analysis fields: this phenomenon can lead to misinterpretations of the calculated correlations between EEG signals. To mitigate this, we employed imaginary coherence, a measure of functional connectivity that is less susceptible to volume conduction effects and provides protection against information leakage between channels.

While our study sample was relatively small, SR‐SE is a rare neurological condition, accounting for less than 10% of SE [3]. Additionally, cyclic seizures are a specific form of SE that remains underexplored. Our database included a high proportion of patients with focal seizures without bilateralization, who were less frequently sedated and intubated for seizure control; thus, increasing the likelihood of movement due to confusion and epileptic motor symptoms, potentially generating more artifacts. Finally, we observed a 33% ICU mortality rate in our sample, which is consistent with those reported in the literature [27].

In this study, LF iCOH between pairs of EEG signals was applied to identify changes in functional connectivity during the ictal phases of cyclic seizures. The results showed a significant increase in synchronization during the final phase of seizure at the regional and electrode levels in patients with bilateralization of seizures and in one patient with focal seizures without bilateralization. However, terminal hypersynchronization was not observed in two patients with localized focal seizures. We also found various topographies and distributions of hypersynchronization. Regional analysis of LF iCOH showed that the increase in synchronization was localized in the anterior and centrotemporal regions, and never in the posterior region. These results were consistent at the electrode level, showing that frontopolar, central and temporal electrodes were more affected by terminal hypersynchronization than occipital electrodes.

Our results suggest a common mechanism of seizure termination across the continuum of “single” seizures to SR‐SE. The diverse topographies of terminal hypersynchronization observed, suggest that this process may not be limited to a specific scalp area nor directly related to the seizure onset zone. Further research is needed to more precisely evaluate the setup of increased synchronization with higher‐density EEG data for a better comprehension of seizure termination mechanisms. Furthermore, hypersynchronization patterns hold potential as EEG biomarkers. Analyzing the spatial distribution of terminal hypersynchronization could help to target specific brain areas for neuromodulation therapies or to assess the efficacy of antiseizure medications during status epilepticus.

## Author Contributions

**Mario Chavez:** conceptualization, methodology, supervision, data curation, formal analysis, writing – review and editing. **Virginie Lambrecq:** conceptualization, methodology, supervision, writing – review and editing. **Sophie Demeret:** resources, writing – review and editing. **Gaspard Martet:** formal analysis, writing – review and editing, visualization. **Maeva Le Goïc:** data curation, writing – review and editing. **Alexandre Ledos:** conceptualization, data curation, methodology, formal analysis, writing – original draft, writing – review and editing.

## Ethics Statement

This research project was conducted in accordance with French regulations and with the Declaration of Helsinki. We confirm that we have read the Journal's position on issues involved in ethical publication and affirm that this report is consistent with those guidelines.

## Conflicts of Interest

The authors declare no conflicts of interest.

## Supporting information


**Appendix S1:** ene70428‐sup‐0001‐AppendixS1.pdf.

## Data Availability

The data that support the findings of this study are available from the corresponding author upon reasonable request.
