# Supplementary material for: Spatiotemporal Brain Dynamics During Cyclic Seizures of Super‐Refractory Status Epilepticus
Source: Eur J Neurol. 2025 Nov 26;32(11):e70428. doi: 10.1111/ene.70428 (PMC12658284; doi:10.1111/ene.70428)
Supplement: Supplementary file 1 — Appendix S1: ene70428‐sup‐0001‐AppendixS1.pdf. [file ENE-32-e70428-s001.pdf]

Supplementary

2025-04-29

Contents

1 Regions analysis **2**
1.1 Model 1: including all patients, natural logarithm transformation . . . 2
1.2 Model 2: Patient 1, natural logarithm transformation . . . 4
1.3 Model 3: Patient 2, natural logarithm transformation . . . 6
1.4 Model 4: Patient 3, natural logarithm transformation . . . 8
1.5 Model 5: Patient 4, natural logarithm transformation . . . 10
1.6 Model 6: Patient 5, natural logarithm transformation . . . 12
1.7 Model 7: Patient 6, natural logarithm transformation . . . 14
1.8 Model 8: Patient 7, natural logarithm transformation . . . 16
1.9 Model 9: Patient 8, natural logarithm transformation . . . 18
1.10 Model 10: Patient 9, natural logarithm transformation . . . 20
1.11 Model 11: Patient 10, natural logarithm transformation . . . 22

2 Electrodes analysis **24**
2.1 Model 1: including all patients, square-root transformation . . . 24
2.2 Model 2: Patient 1, no transformation . . . 26
2.3 Model 3: Patient 2, no transformation . . . 28
2.4 Model 4: Patient 3, no transformation . . . 30
2.5 Model 5: Patient 4, no transformation . . . 32
2.6 Model 6: Patient 5, no transformation . . . 34
2.7 Model 7: Patient 6, no transformation . . . 36
2.8 Model 8: Patient 7, no transformation . . . 38
2.9 Model 9: Patient 8, no transformation . . . 40
2.10 Model 10: Patient 9, no transformation . . . 42
2.11 Model 11: Patient 10, no transformation . . . 44

Where appropriate, modeling was performed to transformed data (using natural logarithm or square-root) to better meet the normality assumption of residuals.

1 Regions analysis

Differences in ICOH were studied for the three states (Precrit, CritB, Postcrit) with critE within the five regions (Anterior, Posterior, Central, Left, Right). The tests were performed using linear mixed effect models for each patient (n = 10) and a global model including all patients.

1.1 Model 1: including all patients, natural logarithm transformation

1.1.1 Type II Wald Chi-square tests

	Chisq	Df	Pr(>Chisq)	signif
States	882.2624	3	< 0.0001	***
Regions	3189.4504	4	< 0.0001	***
States:Regions	139.5370	12	< 0.0001	***

1.1.2 Posthoc emmeans comparisons with Dunnett adjustment against terminal ictal phase

Contrast	Region	Estimate	SE	df	z ratio	p-value	signif
Precrit - CritE	Anterior	0.0204	0.0107	Inf	1.8966	0.1477	ns
CritB - CritE	Anterior	0.1546	0.0107	Inf	14.3928	< 0.0001	***
Postcrit - CritE	Anterior	-0.0218	0.0107	Inf	-2.0255	0.1117	ns
Precrit - CritE	Central	0.0511	0.0107	Inf	4.7546	< 0.0001	***
CritB - CritE	Central	0.1571	0.0107	Inf	14.6336	< 0.0001	***
Postcrit - CritE	Central	-0.0186	0.0107	Inf	-1.7344	0.2044	ns
Precrit - CritE	Posterior	0.0360	0.0107	Inf	3.3535	0.002331	**
CritB - CritE	Posterior	0.1004	0.0107	Inf	9.3522	< 0.0001	***
Postcrit - CritE	Posterior	-0.0004	0.0107	Inf	-0.0329	0.9996	ns
Precrit - CritE	Left	-0.0119	0.0107	Inf	-1.1039	0.5445	ns
CritB - CritE	Left	0.0295	0.0107	Inf	2.7508	0.01683	*
Postcrit - CritE	Left	-0.0332	0.0107	Inf	-3.0890	0.005802	**
Precrit - CritE	Right	-0.0108	0.0107	Inf	-1.0026	0.6102	ns
CritB - CritE	Right	0.1041	0.0107	Inf	9.6948	< 0.0001	***
Postcrit - CritE	Right	-0.0462	0.0107	Inf	-4.3038	< 0.0001	***

1.1.3 Linear mixed effects model diagnostics: Residual plot, Q-Q Plot, Index Plot, Histogram.

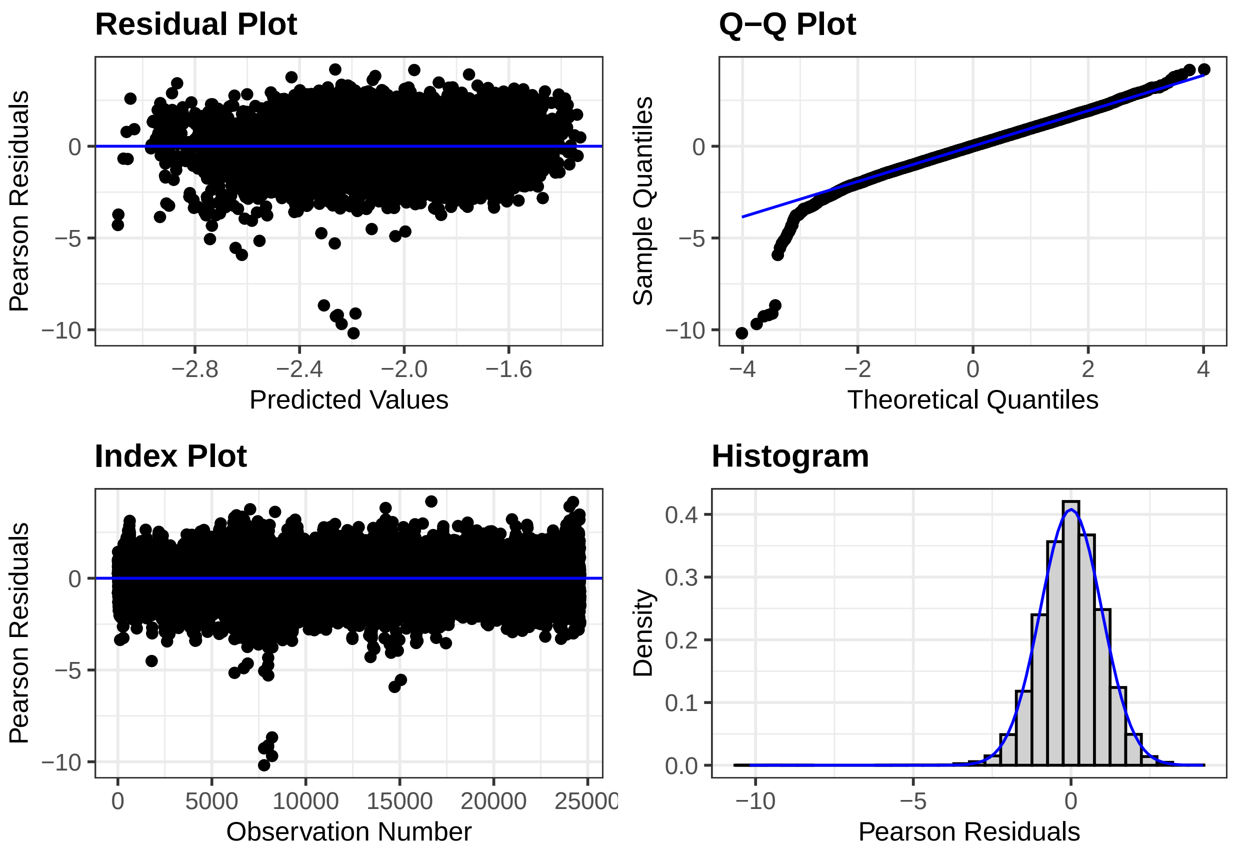

1.2 Model 2: Patient 1, natural logarithm transformation

1.2.1 Type II Wald Chi-square tests

	Chisq	Df	Pr(>Chisq)	signif
States	745.1928	3	< 0.0001	***
Regions	856.5472	4	< 0.0001	***
States:Regions	43.0720	12	< 0.0001	***

1.2.2 Posthoc emmeans comparisons with Dunnett adjustment against terminal ictal phase

Contrast	Region	Estimate	SE	df	z ratio	p-value	signif
Precrit - CritE	Anterior	-0.2921	0.0191	Inf	-15.3160	< 0.0001	***
CritB - CritE	Anterior	-0.1116	0.0191	Inf	-5.8521	< 0.0001	***
Postcrit - CritE	Anterior	-0.2347	0.0191	Inf	-12.3064	< 0.0001	***
Precrit - CritE	Central	-0.1865	0.0191	Inf	-9.7793	< 0.0001	***
CritB - CritE	Central	-0.0566	0.0191	Inf	-2.9655	0.008675	**
Postcrit - CritE	Central	-0.1671	0.0191	Inf	-8.7599	< 0.0001	***
Precrit - CritE	Posterior	-0.1362	0.0191	Inf	-7.1437	< 0.0001	***
CritB - CritE	Posterior	-0.0394	0.0191	Inf	-2.0675	0.1016	ns
Postcrit - CritE	Posterior	-0.1332	0.0191	Inf	-6.9812	< 0.0001	***
Precrit - CritE	Left	-0.1855	0.0191	Inf	-9.7245	< 0.0001	***
CritB - CritE	Left	-0.0532	0.0191	Inf	-2.7919	0.01488	*
Postcrit - CritE	Left	-0.1710	0.0191	Inf	-8.9659	< 0.0001	***
Precrit - CritE	Right	-0.2331	0.0191	Inf	-12.2207	< 0.0001	***
CritB - CritE	Right	-0.0971	0.0191	Inf	-5.0900	< 0.0001	***
Postcrit - CritE	Right	-0.1692	0.0191	Inf	-8.8697	< 0.0001	***

1.2.3 Linear mixed effects model diagnostics: Residual plot, Q-Q Plot, Index Plot, Histogram.

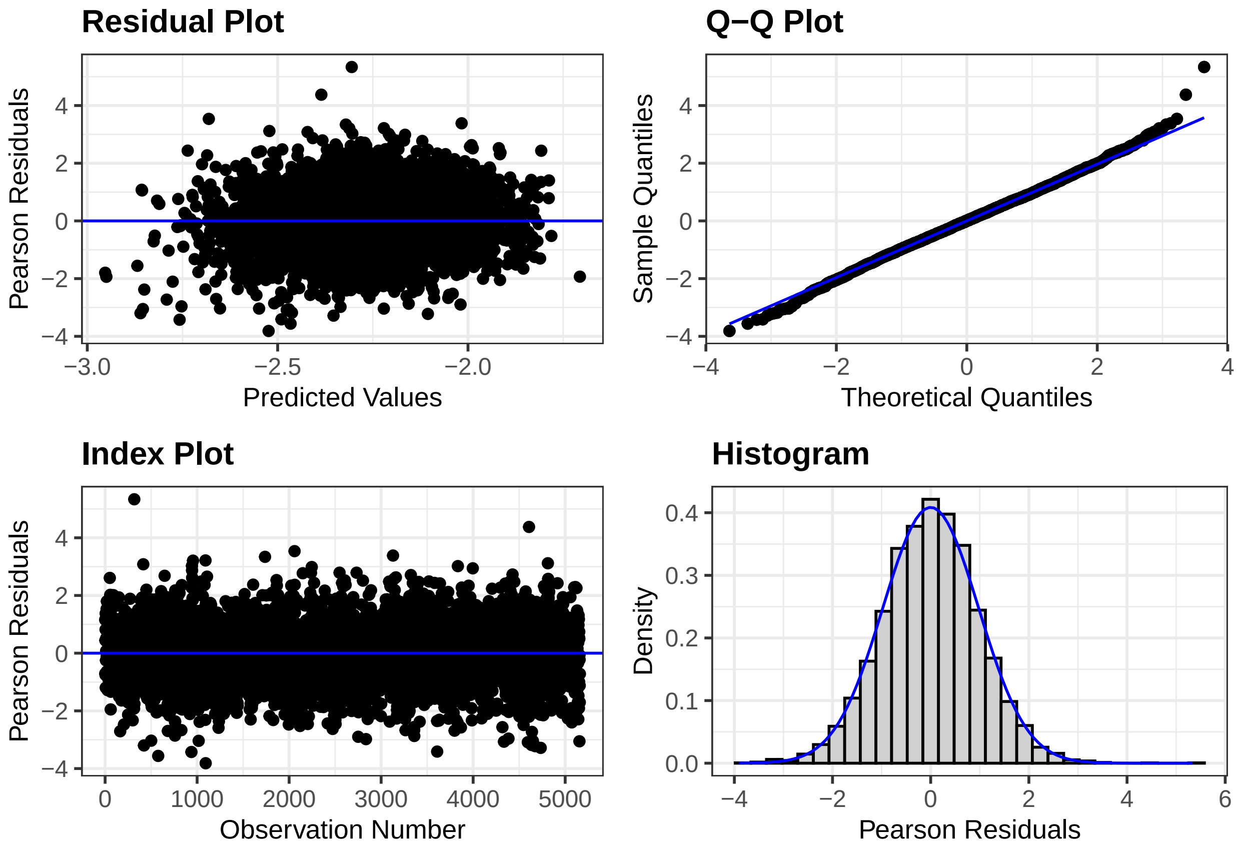

1.3 Model 3: Patient 2, natural logarithm transformation

1.3.1 Type II Wald Chi-square tests

	Chisq	Df	Pr(>Chisq)	signif
States	248.9749	3	< 0.0001	***
Regions	223.2991	4	< 0.0001	***
States:Regions	8.8977	12	0.7116	ns

1.3.2 Posthoc emmeans comparisons with Dunnett adjustment against terminal ictal phase

Contrast	Region	Estimate	SE	df	z ratio	p-value	signif
Precrit - CritE	Anterior	-0.3897	0.0529	1159	-7.3730	< 0.0001	***
CritB - CritE	Anterior	-0.2631	0.0529	1159	-4.9788	< 0.0001	***
Postcrit - CritE	Anterior	-0.2742	0.0529	1159	-5.1887	< 0.0001	***
Precrit - CritE	Central	-0.4222	0.0529	1159	-7.9880	< 0.0001	***
CritB - CritE	Central	-0.2269	0.0529	1159	-4.2928	< 0.0001	***
Postcrit - CritE	Central	-0.3086	0.0529	1159	-5.8381	< 0.0001	***
Precrit - CritE	Posterior	-0.3252	0.0529	1159	-6.1524	< 0.0001	***
CritB - CritE	Posterior	-0.1186	0.0529	1159	-2.2440	0.06735	ns
Postcrit - CritE	Posterior	-0.2133	0.0529	1159	-4.0349	0.0001728	***
Precrit - CritE	Left	-0.3523	0.0529	1159	-6.6650	< 0.0001	***
CritB - CritE	Left	-0.2030	0.0529	1159	-3.8416	0.0003814	***
Postcrit - CritE	Left	-0.2037	0.0529	1159	-3.8533	0.0003639	***
Precrit - CritE	Right	-0.3267	0.0529	1159	-6.1811	< 0.0001	***
CritB - CritE	Right	-0.1404	0.0529	1159	-2.6567	0.02246	*
Postcrit - CritE	Right	-0.2665	0.0529	1159	-5.0425	< 0.0001	***

1.3.3 Linear mixed effects model diagnostics: Residual plot, Q-Q Plot, Index Plot, Histogram.

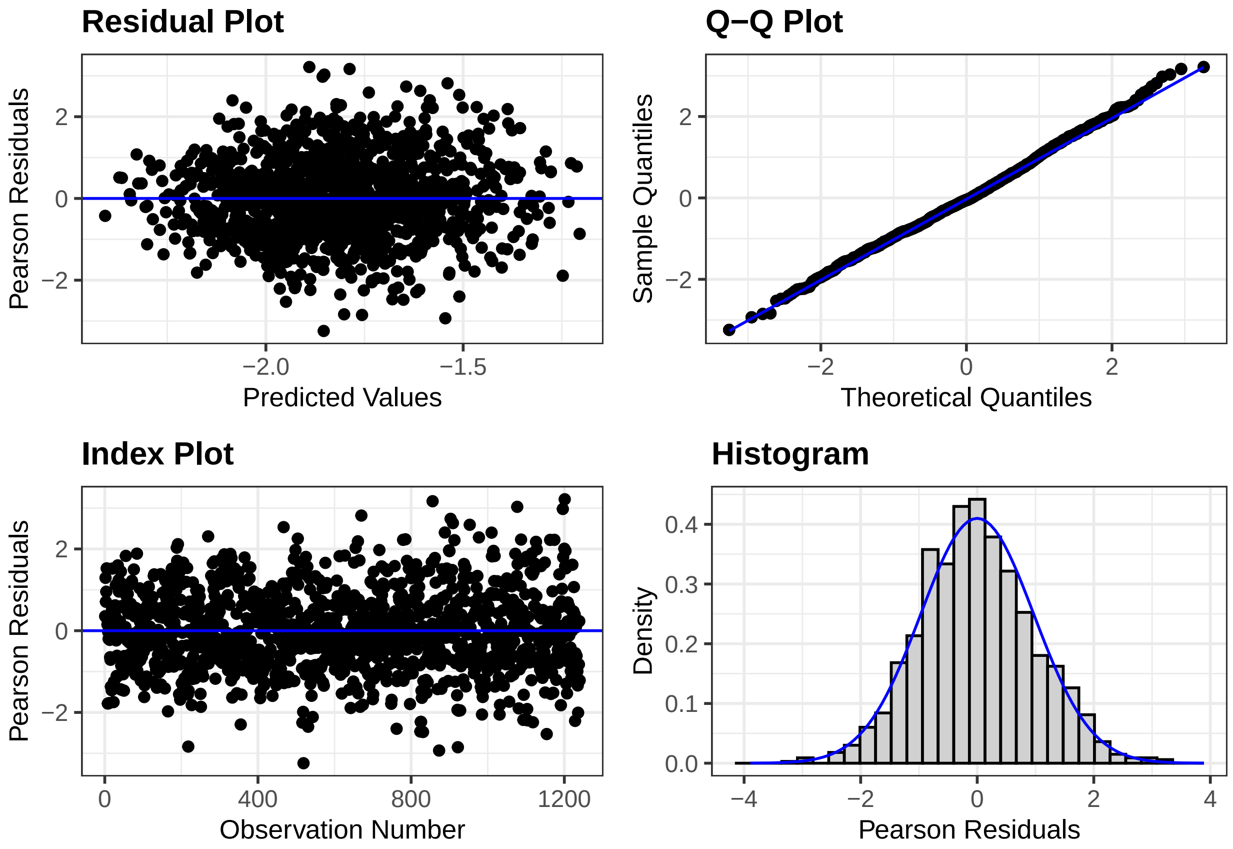

1.4 Model 4: Patient 3, natural logarithm transformation

1.4.1 Type II Wald Chi-square tests

	Chisq	Df	Pr(>Chisq)	signif
States	38.8960	3	< 0.0001	***
Regions	163.2528	4	< 0.0001	***
States:Regions	47.3625	12	< 0.0001	***

1.4.2 Posthoc emmeans comparisons with Dunnett adjustment against terminal ictal phase

Contrast	Region	Estimate	SE	df	z ratio	p-value	signif
Precrit - CritE	Anterior	-0.0988	0.0463	1995	-2.1328	0.08769	ns
CritB - CritE	Anterior	-0.1344	0.0463	1995	-2.9030	0.01068	*
Postcrit - CritE	Anterior	-0.1714	0.0463	1995	-3.7009	0.0006511	***
Precrit - CritE	Central	-0.1267	0.0463	1995	-2.7355	0.01776	*
CritB - CritE	Central	-0.1874	0.0463	1995	-4.0467	0.0001602	***
Postcrit - CritE	Central	-0.3229	0.0463	1995	-6.9728	< 0.0001	***
Precrit - CritE	Posterior	0.0273	0.0463	1995	0.5889	0.8549	ns
CritB - CritE	Posterior	0.0738	0.0463	1995	1.5942	0.2647	ns
Postcrit - CritE	Posterior	-0.0097	0.0463	1995	-0.2101	0.9824	ns
Precrit - CritE	Left	0.0965	0.0463	1995	2.0843	0.09811	ns
CritB - CritE	Left	0.0998	0.0463	1995	2.1545	0.08333	ns
Postcrit - CritE	Left	-0.0277	0.0463	1995	-0.5987	0.85	ns
Precrit - CritE	Right	-0.0473	0.0463	1995	-1.0205	0.5987	ns
CritB - CritE	Right	-0.0867	0.0463	1995	-1.8719	0.1558	ns
Postcrit - CritE	Right	-0.0865	0.0463	1995	-1.8674	0.1572	ns

1.4.3 Linear mixed effects model diagnostics: Residual plot, Q-Q Plot, Index Plot, Histogram.

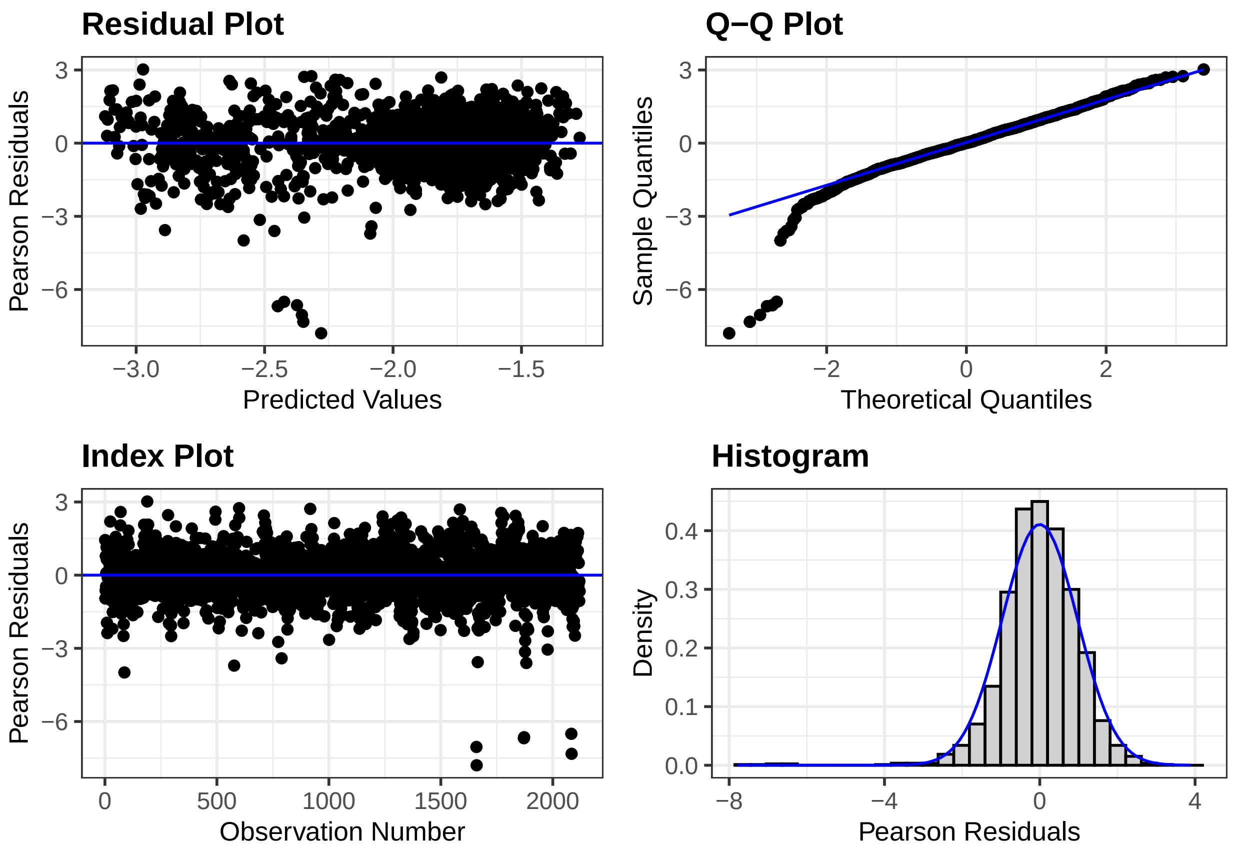

1.5 Model 5: Patient 4, natural logarithm transformation

1.5.1 Type II Wald Chi-square tests

	Chisq	Df	Pr(>Chisq)	signif
States	383.1261	3	< 0.0001	***
Regions	251.0097	4	< 0.0001	***
States:Regions	44.7884	12	< 0.0001	***

1.5.2 Posthoc emmeans comparisons with Dunnett adjustment against terminal ictal phase

Contrast	Region	Estimate	SE	df	z ratio	p-value	signif
Precrit - CritE	Anterior	-0.2596	0.0417	1349	-6.2228	< 0.0001	***
CritB - CritE	Anterior	-0.3515	0.0417	1349	-8.4278	< 0.0001	***
Postcrit - CritE	Anterior	-0.1909	0.0417	1349	-4.5764	< 0.0001	***
Precrit - CritE	Central	-0.3360	0.0417	1349	-8.0558	< 0.0001	***
CritB - CritE	Central	-0.3394	0.0417	1349	-8.1357	< 0.0001	***
Postcrit - CritE	Central	-0.1401	0.0417	1349	-3.3584	0.002353	**
Precrit - CritE	Posterior	-0.2505	0.0417	1349	-6.0049	< 0.0001	***
CritB - CritE	Posterior	-0.1410	0.0417	1349	-3.3801	0.002179	**
Postcrit - CritE	Posterior	0.0962	0.0417	1349	2.3055	0.05776	ns
Precrit - CritE	Left	-0.2751	0.0417	1349	-6.5945	< 0.0001	***
CritB - CritE	Left	-0.2849	0.0417	1349	-6.8302	< 0.0001	***
Postcrit - CritE	Left	-0.0613	0.0417	1349	-1.4693	0.3269	ns
Precrit - CritE	Right	-0.3604	0.0417	1349	-8.6390	< 0.0001	***
CritB - CritE	Right	-0.3497	0.0417	1349	-8.3835	< 0.0001	***
Postcrit - CritE	Right	-0.1475	0.0417	1349	-3.5354	0.001237	**

1.5.3 Linear mixed effects model diagnostics: Residual plot, Q-Q Plot, Index Plot, Histogram.

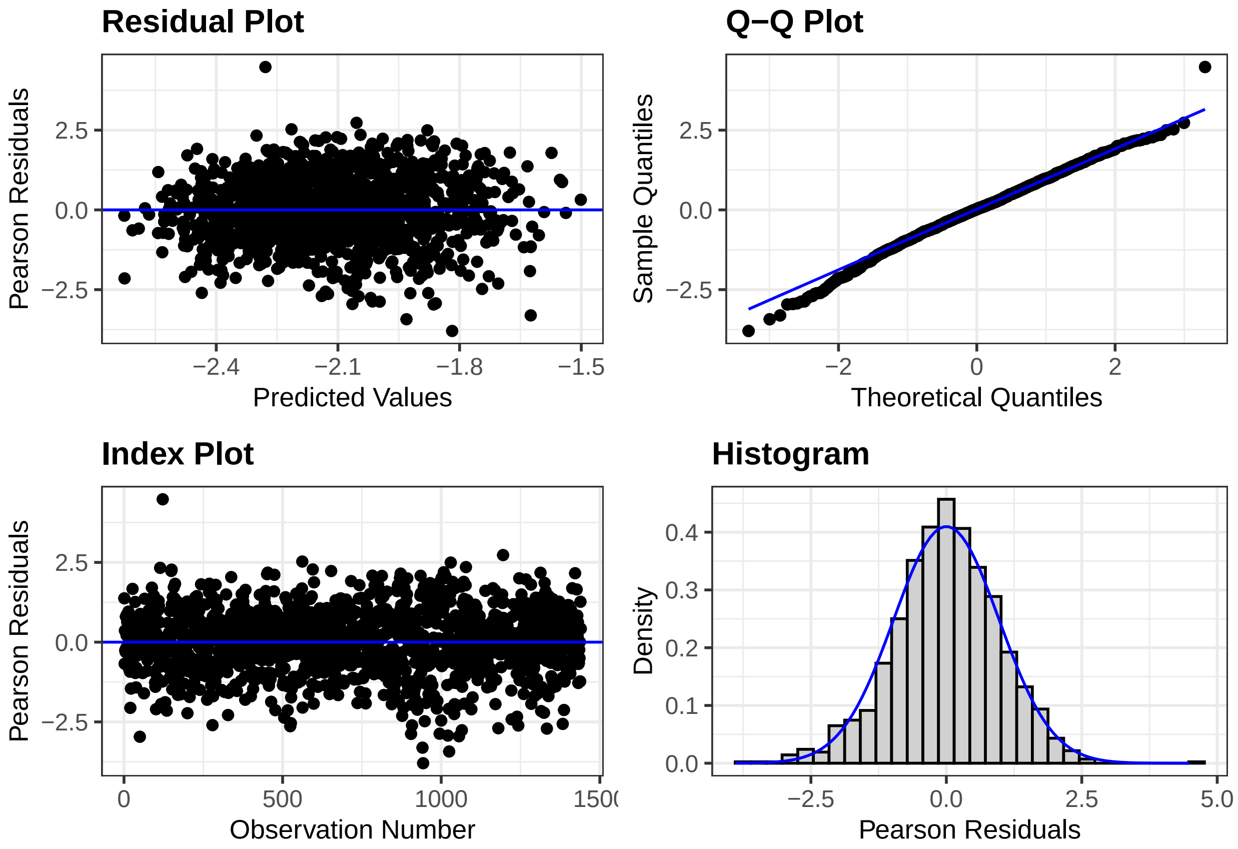

1.6 Model 6: Patient 5, natural logarithm transformation

1.6.1 Type II Wald Chi-square tests

	Chisq	Df	Pr(>Chisq)	signif
States	72.5347	3	< 0.0001	***
Regions	120.6477	4	< 0.0001	***
States:Regions	10.5432	12	0.5684	ns

1.6.2 Posthoc emmeans comparisons with Dunnett adjustment against terminal ictal phase

Contrast	Region	Estimate	SE	df	z ratio	p-value	signif
Precrit - CritE	Anterior	-0.1719	0.0433	1273	-3.9677	0.0002272	***
CritB - CritE	Anterior	-0.1343	0.0433	1273	-3.1003	0.005708	**
Postcrit - CritE	Anterior	-0.0922	0.0433	1273	-2.1283	0.08879	ns
Precrit - CritE	Central	-0.1515	0.0433	1273	-3.4955	0.001436	**
CritB - CritE	Central	-0.0475	0.0433	1273	-1.0956	0.5501	ns
Postcrit - CritE	Central	-0.1419	0.0433	1273	-3.2753	0.003155	**
Precrit - CritE	Posterior	-0.1641	0.0433	1273	-3.7880	0.0004699	***
CritB - CritE	Posterior	-0.0736	0.0433	1273	-1.6990	0.2191	ns
Postcrit - CritE	Posterior	-0.1105	0.0433	1273	-2.5496	0.03033	*
Precrit - CritE	Left	-0.0969	0.0433	1273	-2.2357	0.0687	ns
CritB - CritE	Left	-0.0612	0.0433	1273	-1.4129	0.3573	ns
Postcrit - CritE	Left	-0.0519	0.0433	1273	-1.1967	0.4857	ns
Precrit - CritE	Right	-0.2260	0.0433	1273	-5.2153	< 0.0001	***
CritB - CritE	Right	-0.1337	0.0433	1273	-3.0853	0.005996	**
Postcrit - CritE	Right	-0.1435	0.0433	1273	-3.3122	0.002774	**

1.6.3 Linear mixed effects model diagnostics: Residual plot, Q-Q Plot, Index Plot, Histogram.

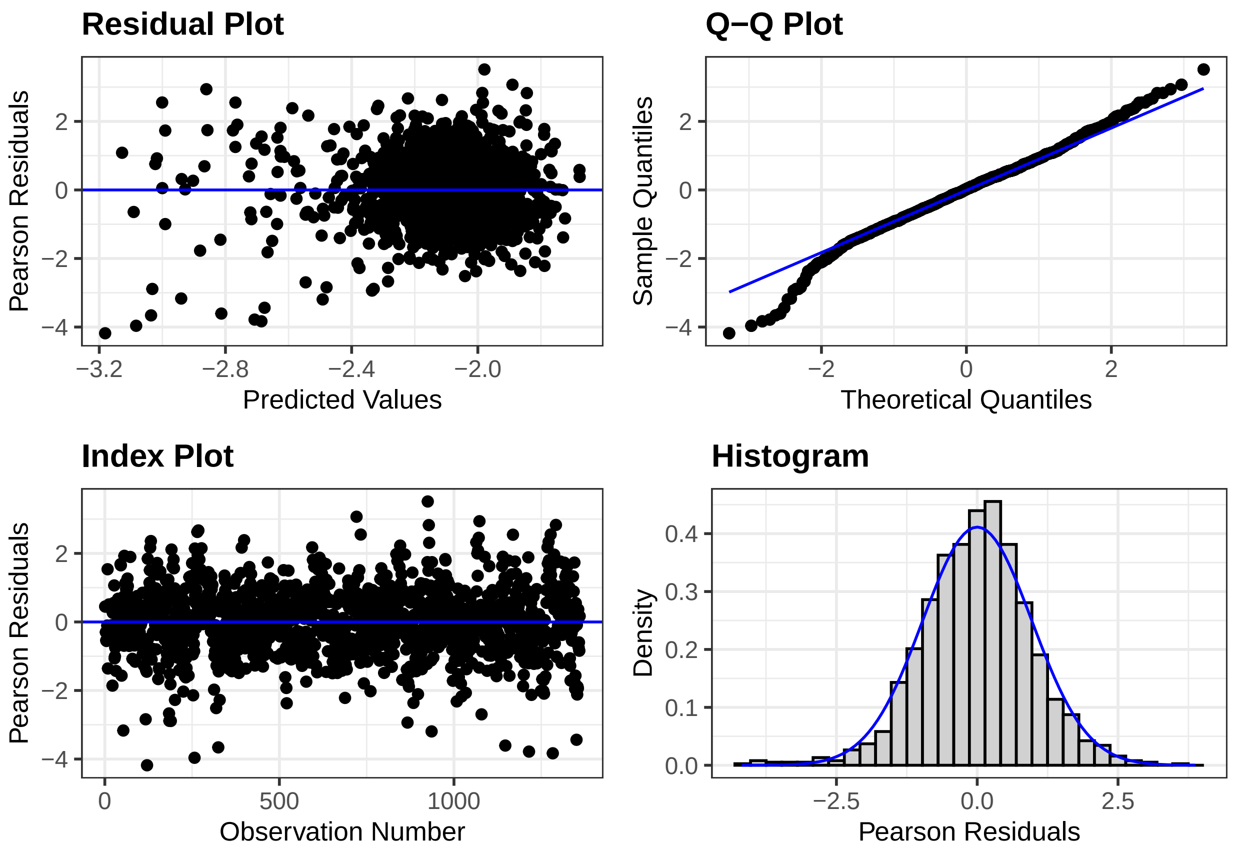

1.7 Model 7: Patient 6, natural logarithm transformation

1.7.1 Type II Wald Chi-square tests

	Chisq	Df	Pr(>Chisq)	signif
States	104.0404	3	< 0.0001	***
Regions	472.0673	4	< 0.0001	***
States:Regions	73.6867	12	< 0.0001	***

1.7.2 Posthoc emmeans comparisons with Dunnett adjustment against terminal ictal phase

Contrast	Region	Estimate	SE	df	z ratio	p-value	signif
Precrit - CritE	Anterior	-0.1863	0.0352	1159	-5.2887	< 0.0001	***
CritB - CritE	Anterior	-0.1479	0.0352	1159	-4.1981	< 0.0001	***
Postcrit - CritE	Anterior	-0.1953	0.0352	1159	-5.5437	< 0.0001	***
Precrit - CritE	Central	-0.0574	0.0352	1159	-1.6308	0.2483	ns
CritB - CritE	Central	-0.1375	0.0352	1159	-3.9041	0.0002963	***
Postcrit - CritE	Central	-0.1467	0.0352	1159	-4.1637	1e-04	***
Precrit - CritE	Posterior	-0.0743	0.0352	1159	-2.1098	0.09272	ns
CritB - CritE	Posterior	-0.0137	0.0352	1159	-0.3901	0.937	ns
Postcrit - CritE	Posterior	-0.0784	0.0352	1159	-2.2253	0.0705	ns
Precrit - CritE	Left	0.0093	0.0352	1159	0.2628	0.9721	ns
CritB - CritE	Left	0.0439	0.0352	1159	1.2461	0.455	ns
Postcrit - CritE	Left	-0.0227	0.0352	1159	-0.6437	0.8271	ns
Precrit - CritE	Right	-0.2925	0.0352	1159	-8.3036	< 0.0001	***
CritB - CritE	Right	-0.2553	0.0352	1159	-7.2490	< 0.0001	***
Postcrit - CritE	Right	-0.3150	0.0352	1159	-8.9437	< 0.0001	***

1.7.3 Linear mixed effects model diagnostics: Residual plot, Q-Q Plot, Index Plot, Histogram.

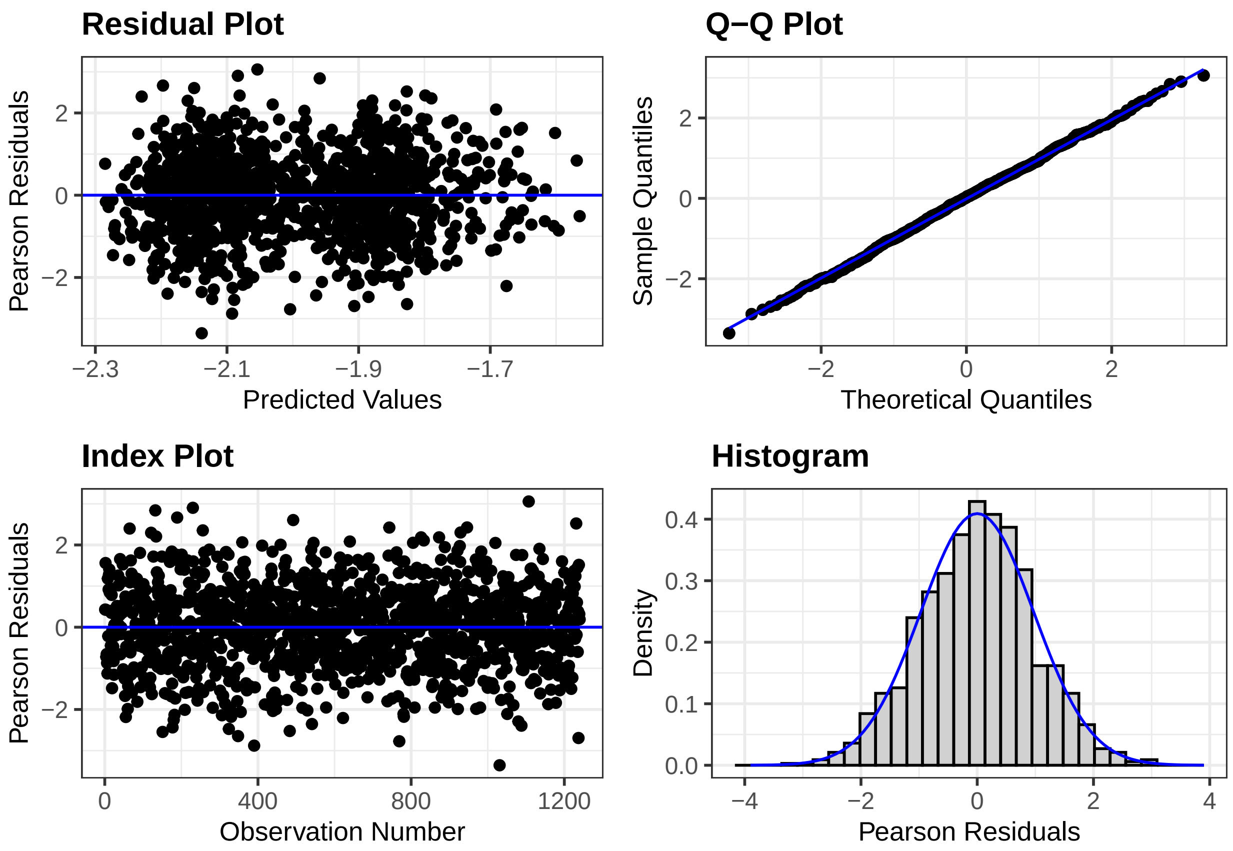

1.8 Model 8: Patient 7, natural logarithm transformation

1.8.1 Type II Wald Chi-square tests

	Chisq	Df	Pr(>Chisq)	signif
States	219.7301	3	< 0.0001	***
Regions	126.4672	4	< 0.0001	***
States:Regions	139.3456	12	< 0.0001	***

1.8.2 Posthoc emmeans comparisons with Dunnett adjustment against terminal ictal phase

Contrast	Region	Estimate	SE	df	z ratio	p-value	signif
Precrit - CritE	Anterior	-0.1359	0.0358	1577	-3.7967	0.00045	***
CritB - CritE	Anterior	-0.0696	0.0358	1577	-1.9432	0.1342	ns
Postcrit - CritE	Anterior	-0.1572	0.0358	1577	-4.3916	< 0.0001	***
Precrit - CritE	Central	-0.0921	0.0358	1577	-2.5741	0.02829	*
CritB - CritE	Central	0.1465	0.0358	1577	4.0918	0.0001336	***
Postcrit - CritE	Central	-0.0989	0.0358	1577	-2.7621	0.01645	*
Precrit - CritE	Posterior	-0.0548	0.0358	1577	-1.5324	0.2945	ns
CritB - CritE	Posterior	0.1819	0.0358	1577	5.0818	< 0.0001	***
Postcrit - CritE	Posterior	-0.1572	0.0358	1577	-4.3915	< 0.0001	***
Precrit - CritE	Left	0.1530	0.0358	1577	4.2741	< 0.0001	***
CritB - CritE	Left	0.3116	0.0358	1577	8.7059	< 0.0001	***
Postcrit - CritE	Left	-0.0144	0.0358	1577	-0.4027	0.9328	ns
Precrit - CritE	Right	-0.2450	0.0358	1577	-6.8453	< 0.0001	***
CritB - CritE	Right	-0.1447	0.0358	1577	-4.0428	0.0001644	***
Postcrit - CritE	Right	-0.2699	0.0358	1577	-7.5400	< 0.0001	***

1.8.3 Linear mixed effects model diagnostics: Residual plot, Q-Q Plot, Index Plot, Histogram.

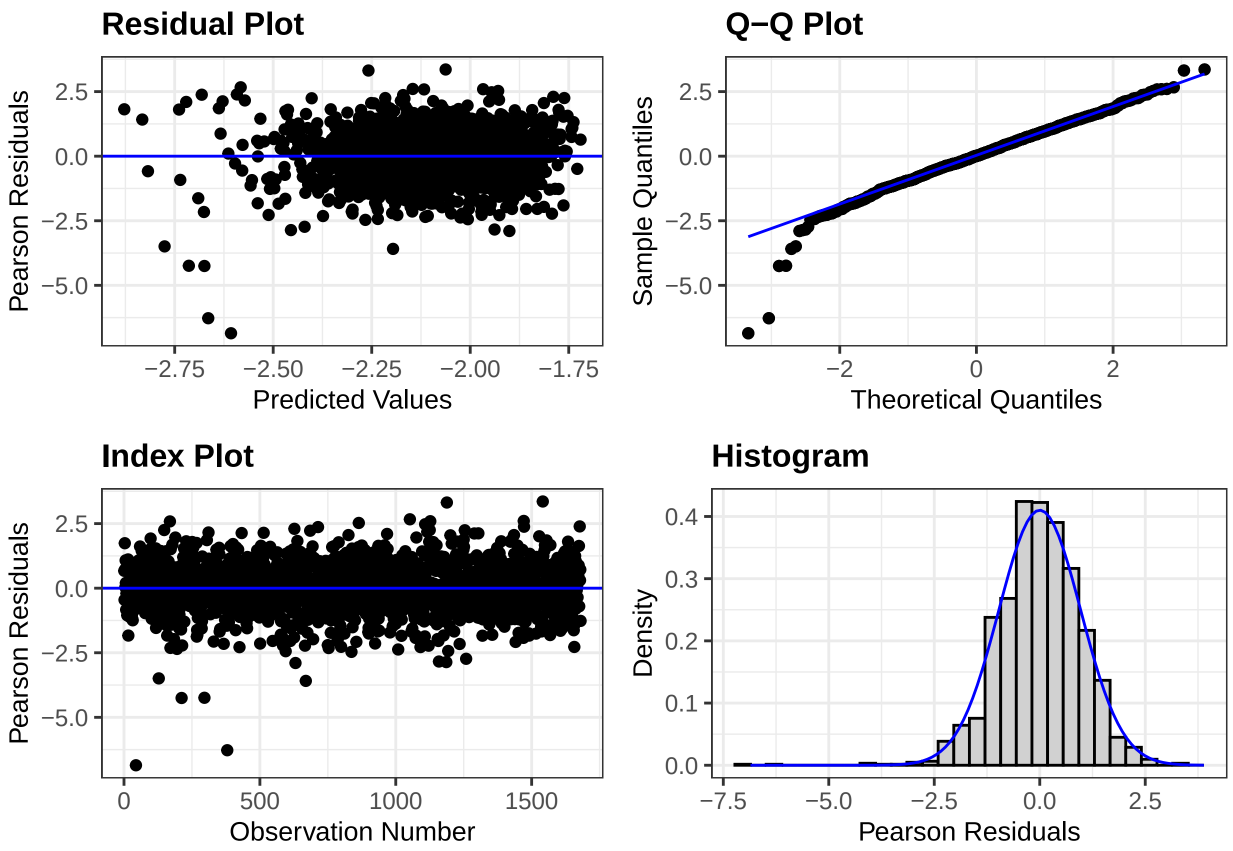

1.9 Model 9: Patient 8, natural logarithm transformation

1.9.1 Type II Wald Chi-square tests

	Chisq	Df	Pr(>Chisq)	signif
States	515.7253	3	< 0.0001	***
Regions	5405.4545	4	< 0.0001	***
States:Regions	416.3480	12	< 0.0001	***

1.9.2 Posthoc emmeans comparisons with Dunnett adjustment against terminal ictal phase

Contrast	Region	Estimate	SE	df	z ratio	p-value	signif
Precrit - CritE	Anterior	-0.1902	0.0169	Inf	-11.2287	< 0.0001	***
CritB - CritE	Anterior	-0.1846	0.0169	Inf	-10.8941	< 0.0001	***
Postcrit - CritE	Anterior	-0.1355	0.0169	Inf	-7.9999	< 0.0001	***
Precrit - CritE	Central	-0.2900	0.0169	Inf	-17.1146	< 0.0001	***
CritB - CritE	Central	-0.2074	0.0169	Inf	-12.2429	< 0.0001	***
Postcrit - CritE	Central	-0.1906	0.0169	Inf	-11.2466	< 0.0001	***
Precrit - CritE	Posterior	0.0640	0.0169	Inf	3.7749	0.0004733	***
CritB - CritE	Posterior	-0.1180	0.0169	Inf	-6.9646	< 0.0001	***
Postcrit - CritE	Posterior	0.1187	0.0169	Inf	7.0083	< 0.0001	***
Precrit - CritE	Left	-0.1672	0.0169	Inf	-9.8694	< 0.0001	***
CritB - CritE	Left	-0.2112	0.0169	Inf	-12.4663	< 0.0001	***
Postcrit - CritE	Left	-0.1547	0.0169	Inf	-9.1312	< 0.0001	***
Precrit - CritE	Right	-0.0238	0.0169	Inf	-1.4044	0.3617	ns
CritB - CritE	Right	-0.0846	0.0169	Inf	-4.9926	< 0.0001	***
Postcrit - CritE	Right	0.0442	0.0169	Inf	2.6106	0.02531	*

1.9.3 Linear mixed effects model diagnostics: Residual plot, Q-Q Plot, Index Plot, Histogram.

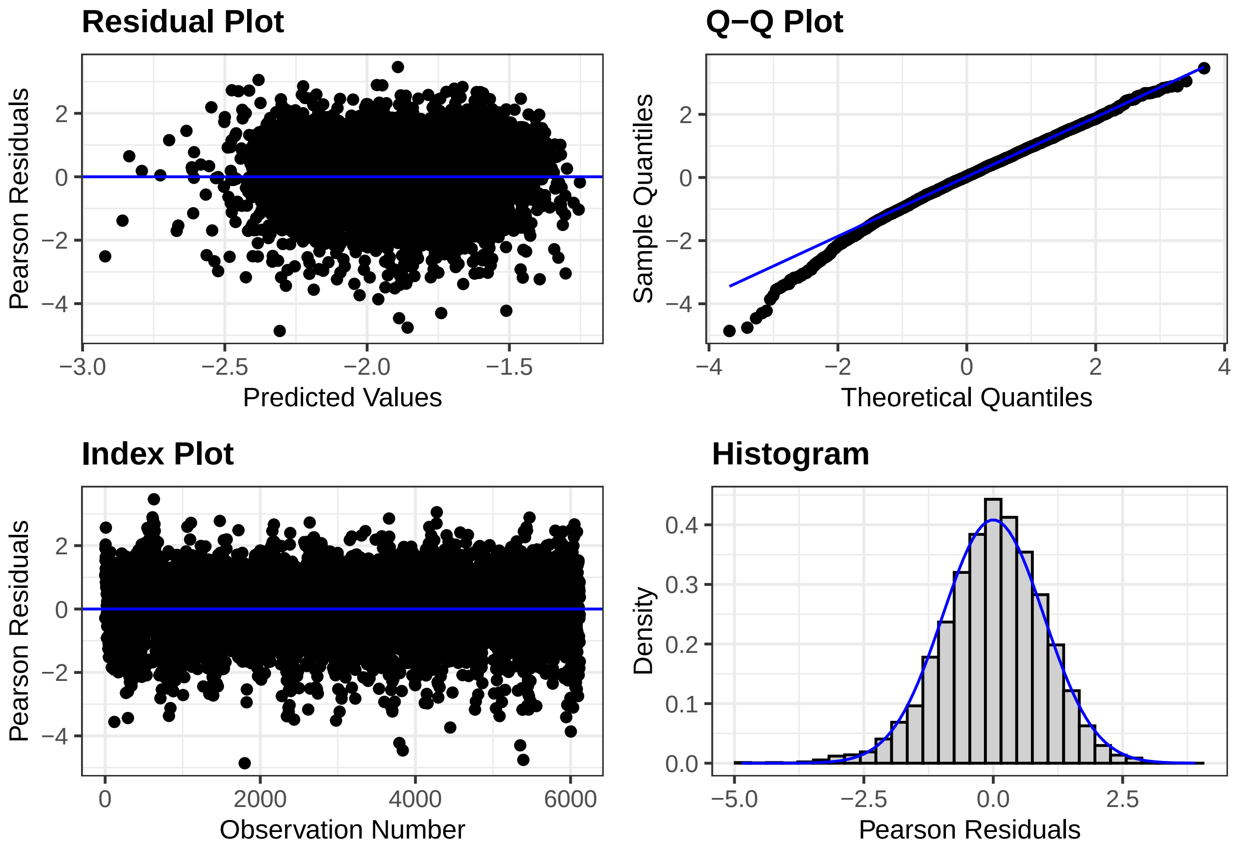

1.10 Model 10: Patient 9, natural logarithm transformation

1.10.1 Type II Wald Chi-square tests

	Chisq	Df	Pr(>Chisq)	signif
States	44.4889	3	< 0.0001	***
Regions	1868.8988	4	< 0.0001	***
States:Regions	5.2972	12	0.9473	ns

1.10.2 Posthoc emmeans comparisons with Dunnett adjustment against terminal ictal phase

Contrast	Region	Estimate	SE	df	z ratio	p-value	signif
Precrit - CritE	Anterior	0.0422	0.0273	2261	1.5453	0.288	ns
CritB - CritE	Anterior	0.0275	0.0273	2261	1.0067	0.6076	ns
Postcrit - CritE	Anterior	-0.0585	0.0273	2261	-2.1422	0.08576	ns
Precrit - CritE	Central	0.0311	0.0273	2261	1.1378	0.5229	ns
CritB - CritE	Central	0.0131	0.0273	2261	0.4786	0.9044	ns
Postcrit - CritE	Central	-0.0375	0.0273	2261	-1.3726	0.3798	ns
Precrit - CritE	Posterior	0.0064	0.0273	2261	0.2351	0.9779	ns
CritB - CritE	Posterior	0.0358	0.0273	2261	1.3117	0.4152	ns
Postcrit - CritE	Posterior	-0.0338	0.0273	2261	-1.2396	0.4589	ns
Precrit - CritE	Left	0.0113	0.0273	2261	0.4130	0.9292	ns
CritB - CritE	Left	0.0055	0.0273	2261	0.2006	0.984	ns
Postcrit - CritE	Left	-0.0430	0.0273	2261	-1.5753	0.2736	ns
Precrit - CritE	Right	0.0310	0.0273	2261	1.1336	0.5256	ns
CritB - CritE	Right	0.0329	0.0273	2261	1.2031	0.4815	ns
Postcrit - CritE	Right	-0.0615	0.0273	2261	-2.2519	0.06584	ns

1.10.3 Linear mixed effects model diagnostics: Residual plot, Q-Q Plot, Index Plot, Histogram.

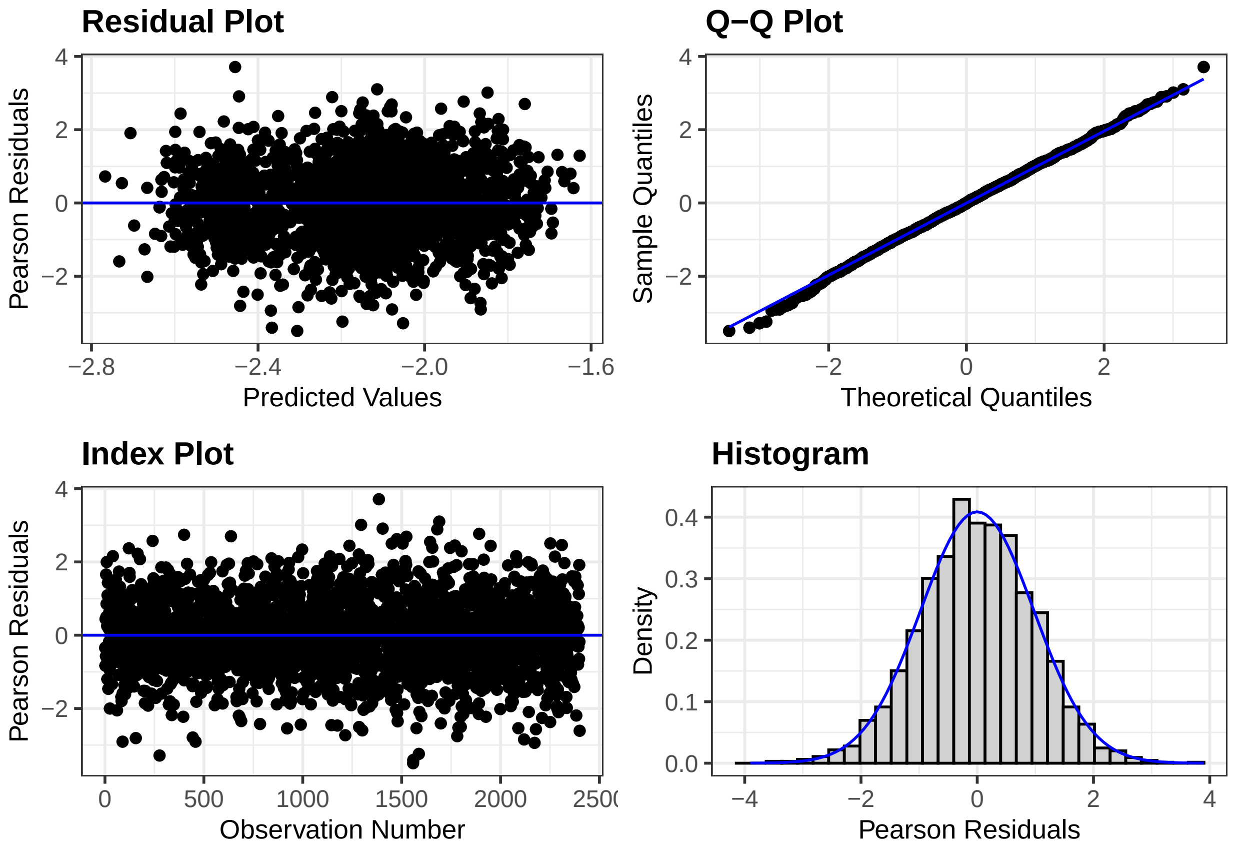

1.11 Model 11: Patient 10, natural logarithm transformation

1.11.1 Type II Wald Chi-square tests

	Chisq	Df	Pr(>Chisq)	signif
States	30.8551	3	< 0.0001	***
Regions	1586.2105	4	< 0.0001	***
States:Regions	46.1879	12	< 0.0001	***

1.11.2 Posthoc emmeans comparisons with Dunnett adjustment against terminal ictal phase

Contrast	Region	Estimate	SE	df	z ratio	p-value	signif
Precrit - CritE	Anterior	-0.0040	0.0288	1729	-0.1375	0.9927	ns
CritB - CritE	Anterior	-0.0332	0.0288	1729	-1.1515	0.5142	ns
Postcrit - CritE	Anterior	-0.0060	0.0288	1729	-0.2098	0.9825	ns
Precrit - CritE	Central	0.0253	0.0288	1729	0.8792	0.6894	ns
CritB - CritE	Central	0.0416	0.0288	1729	1.4455	0.3394	ns
Postcrit - CritE	Central	0.0339	0.0288	1729	1.1776	0.4977	ns
Precrit - CritE	Posterior	-0.0722	0.0288	1729	-2.5068	0.03403	*
CritB - CritE	Posterior	-0.0943	0.0288	1729	-3.2726	0.003163	**
Postcrit - CritE	Posterior	-0.0147	0.0288	1729	-0.5091	0.8916	ns
Precrit - CritE	Left	-0.0187	0.0288	1729	-0.6479	0.8249	ns
CritB - CritE	Left	-0.0405	0.0288	1729	-1.4064	0.3608	ns
Postcrit - CritE	Left	-0.0085	0.0288	1729	-0.2957	0.9644	ns
Precrit - CritE	Right	-0.1729	0.0288	1729	-6.0019	< 0.0001	***
CritB - CritE	Right	-0.1539	0.0288	1729	-5.3416	< 0.0001	***
Postcrit - CritE	Right	-0.0249	0.0288	1729	-0.8653	0.6981	ns

1.11.3 Linear mixed effects model diagnostics: Residual plot, Q-Q Plot, Index Plot, Histogram.

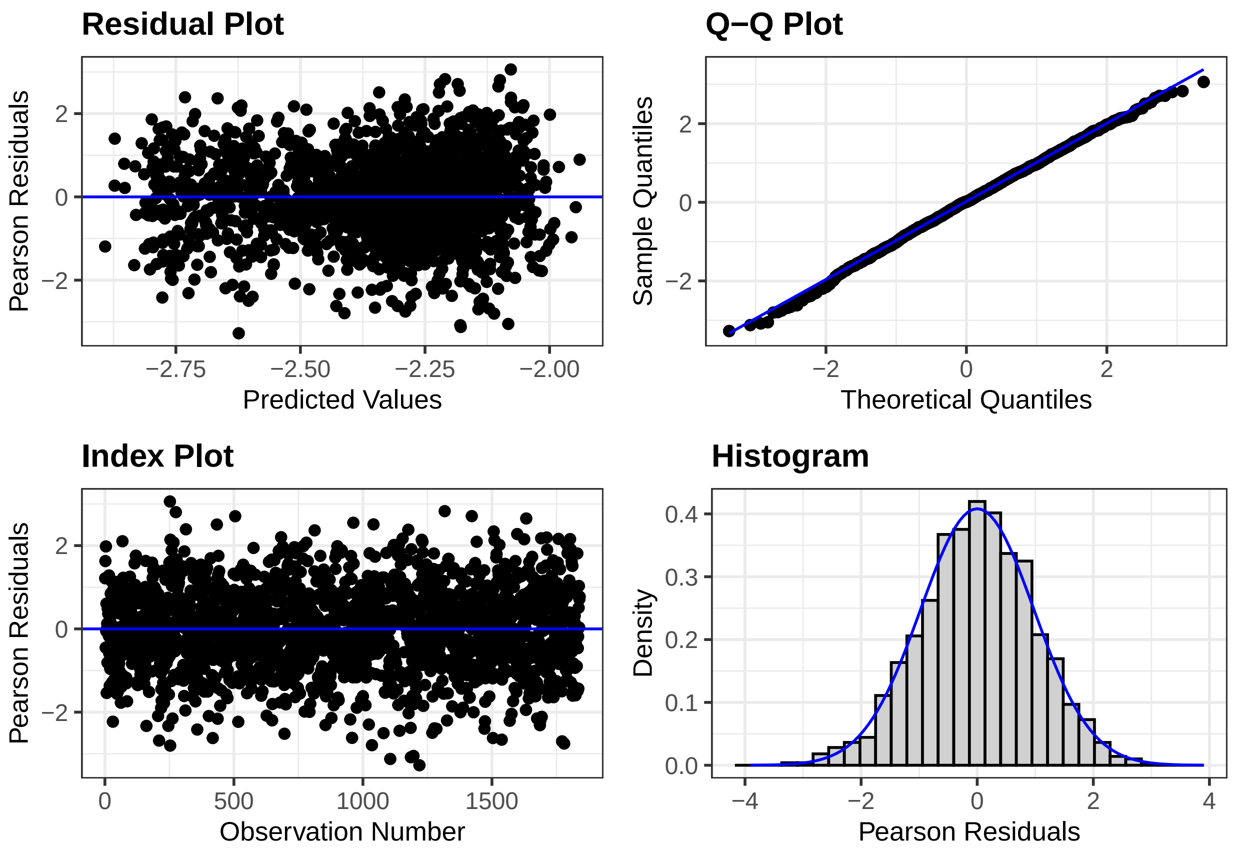

2 Electrodes analysis

Differences in ICOH were studied for the three states (Precrit, CritB, Postcrit) with critE within the eight electrodes (Fp2, Fp1, C4, T4, O2, C3, T3, O1). The tests were performed using linear mixed effect models for each patient (n = 10) and a global model including all patients.

2.1 Model 1: including all patients, square-root transformation

2.1.1 Type II Wald Chi-square tests

	Chisq	Df	Pr(>Chisq)	signif
States	739.6409	3	< 0.0001	***
Electrodes	107.2955	7	< 0.0001	***
States:Electrodes	69.0060	21	< 0.0001	***

2.1.2 Posthoc emmeans comparisons with Dunnett adjustment against terminal ictal phase

Contrast	Region	Estimate	SE	df	z ratio	p-value	signif
Precrit - CritE	Fp2	0.1266	0.0357	Inf	3.5506	0.00113	**
CritB - CritE	Fp2	0.3262	0.0340	Inf	9.6000	< 0.0001	***
Postcrit - CritE	Fp2	0.0772	0.0369	Inf	2.0921	0.09608	ns
Precrit - CritE	C4	0.0548	0.0364	Inf	1.5071	0.3069	ns
CritB - CritE	C4	0.3001	0.0341	Inf	8.8040	< 0.0001	***
Postcrit - CritE	C4	0.0048	0.0378	Inf	0.1261	0.9938	ns
Precrit - CritE	T4	0.0909	0.0374	Inf	2.4317	0.04137	*
CritB - CritE	T4	0.3281	0.0350	Inf	9.3812	< 0.0001	***
Postcrit - CritE	T4	-0.0002	0.0374	Inf	-0.0056	1	ns
Precrit - CritE	O2	-0.0727	0.0371	Inf	-1.9585	0.1295	ns
CritB - CritE	O2	0.3255	0.0346	Inf	9.4024	< 0.0001	***
Postcrit - CritE	O2	-0.0297	0.0385	Inf	-0.7728	0.7545	ns
Precrit - CritE	Fp1	0.1060	0.0374	Inf	2.8369	0.01297	*
CritB - CritE	Fp1	0.2220	0.0354	Inf	6.2652	< 0.0001	***
Postcrit - CritE	Fp1	0.0597	0.0374	Inf	1.5957	0.2637	ns
Precrit - CritE	C3	0.1310	0.0376	Inf	3.4884	0.001426	**
CritB - CritE	C3	0.2402	0.0351	Inf	6.8500	< 0.0001	***
Postcrit - CritE	C3	0.0258	0.0386	Inf	0.6693	0.8134	ns
Precrit - CritE	T3	0.1373	0.0361	Inf	3.8034	0.0004222	***
CritB - CritE	T3	0.3121	0.0339	Inf	9.2079	< 0.0001	***
Postcrit - CritE	T3	0.0250	0.0374	Inf	0.6675	0.8143	ns
Precrit - CritE	O1	-0.0059	0.0381	Inf	-0.1553	0.9906	ns
CritB - CritE	O1	0.3048	0.0349	Inf	8.7304	< 0.0001	***
Postcrit - CritE	O1	-0.0190	0.0389	Inf	-0.4881	0.9005	ns

2.1.3 Linear mixed effects model diagnostics: Residual plot, Q-Q Plot, Index Plot, Histogram.

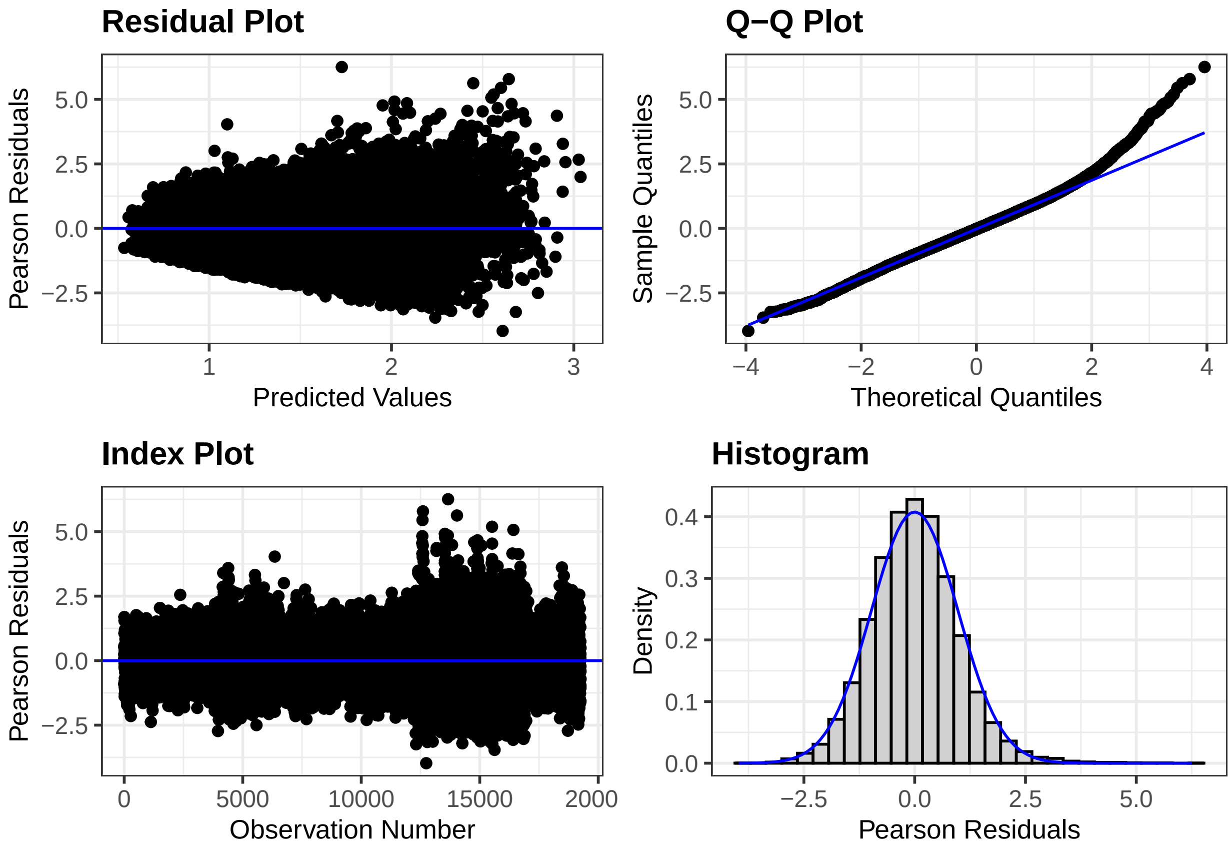

2.2 Model 2: Patient 1, no transformation

2.2.1 Type II Wald Chi-square tests

	Chisq	Df	Pr(>Chisq)	signif
States	1398.9774	3	< 0.0001	***
Electrodes	30.7799	7	< 0.0001	***
States:Electrodes	63.1333	21	< 0.0001	***

2.2.2 Posthoc emmeans comparisons with Dunnett adjustment against terminal ictal phase

Contrast	Region	Estimate	SE	df	z ratio	p-value	signif
Precrit - CritE	Fp2	-6.5094	0.3897	Inf	-16.7025	< 0.0001	***
CritB - CritE	Fp2	-3.5782	0.3897	Inf	-9.1812	< 0.0001	***
Postcrit - CritE	Fp2	-5.5391	0.3897	Inf	-14.2126	< 0.0001	***
Precrit - CritE	C4	-4.1669	0.3897	Inf	-10.6917	< 0.0001	***
CritB - CritE	C4	-1.6848	0.3897	Inf	-4.3231	< 0.0001	***
Postcrit - CritE	C4	-3.5361	0.3897	Inf	-9.0732	< 0.0001	***
Precrit - CritE	T4	-3.9765	0.3897	Inf	-10.2033	< 0.0001	***
CritB - CritE	T4	-2.1008	0.3897	Inf	-5.3905	< 0.0001	***
Postcrit - CritE	T4	-3.6613	0.3897	Inf	-9.3944	< 0.0001	***
Precrit - CritE	O2	-4.0710	0.3897	Inf	-10.4456	< 0.0001	***
CritB - CritE	O2	-1.8295	0.3897	Inf	-4.6942	< 0.0001	***
Postcrit - CritE	O2	-3.6559	0.3897	Inf	-9.3805	< 0.0001	***
Precrit - CritE	Fp1	-5.7529	0.3897	Inf	-14.7613	< 0.0001	***
CritB - CritE	Fp1	-2.4084	0.3897	Inf	-6.1797	< 0.0001	***
Postcrit - CritE	Fp1	-5.4271	0.3897	Inf	-13.9253	< 0.0001	***
Precrit - CritE	C3	-4.0451	0.3897	Inf	-10.3793	< 0.0001	***
CritB - CritE	C3	-2.2577	0.3897	Inf	-5.7931	< 0.0001	***
Postcrit - CritE	C3	-3.7903	0.3897	Inf	-9.7255	< 0.0001	***
Precrit - CritE	T3	-4.1192	0.3897	Inf	-10.5693	< 0.0001	***
CritB - CritE	T3	-1.4664	0.3897	Inf	-3.7625	0.0004972	***
Postcrit - CritE	T3	-4.0100	0.3897	Inf	-10.2892	< 0.0001	***
Precrit - CritE	O1	-3.8234	0.3897	Inf	-9.8104	< 0.0001	***
CritB - CritE	O1	-1.7661	0.3897	Inf	-4.5315	< 0.0001	***
Postcrit - CritE	O1	-3.9067	0.3897	Inf	-10.0240	< 0.0001	***

2.2.3 Linear mixed effects model diagnostics: Residual plot, Q-Q Plot, Index Plot, Histogram.

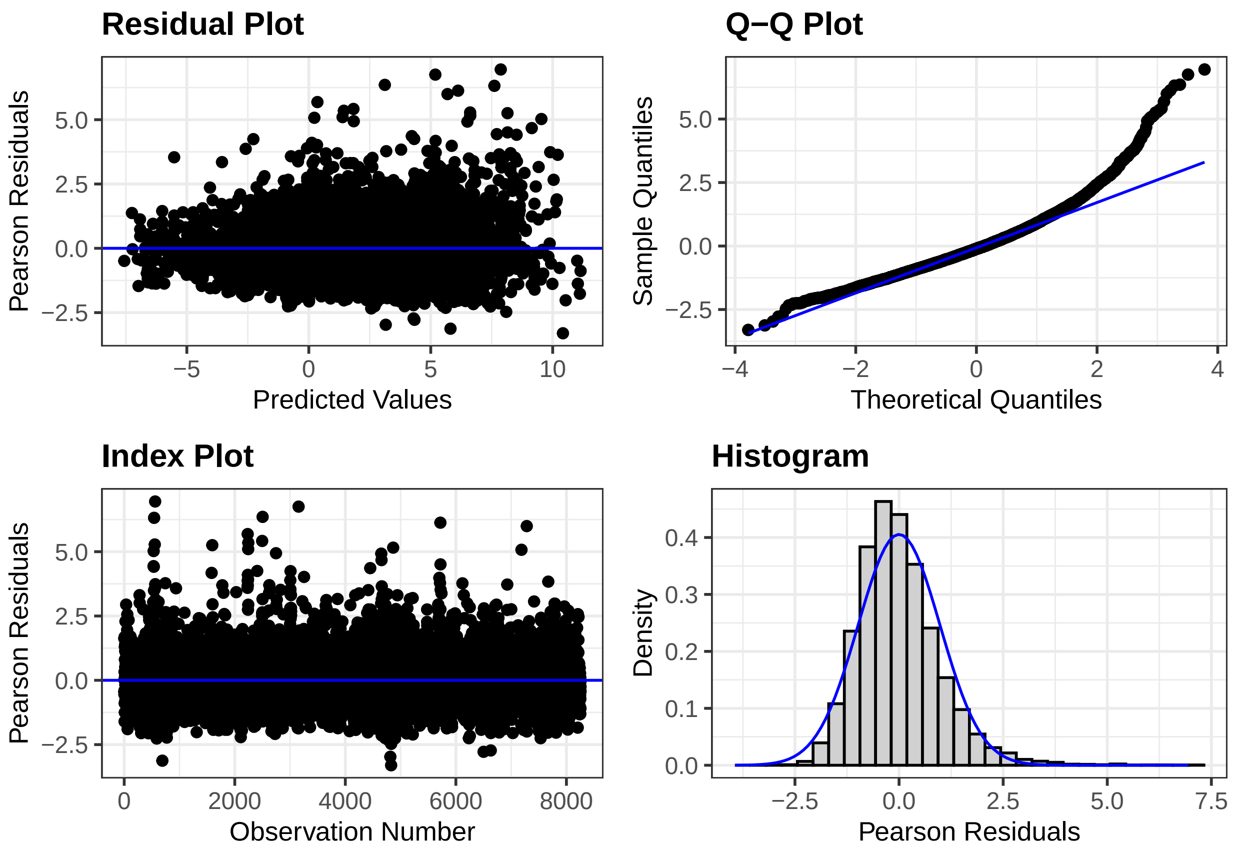

2.3 Model 3: Patient 2, no transformation

2.3.1 Type II Wald Chi-square tests

	Chisq	Df	Pr(>Chisq)	signif
States	565.8314	3	< 0.0001	***
Electrodes	20.9811	7	0.003798	**
States:Electrodes	25.6179	21	0.2214	ns

2.3.2 Posthoc emmeans comparisons with Dunnett adjustment against terminal ictal phase

Contrast	Region	Estimate	SE	df	z ratio	p-value	signif
Precrit - CritE	Fp2	-3.2289	0.4175	1891	-7.7346	< 0.0001	***
CritB - CritE	Fp2	-2.3961	0.4175	1891	-5.7398	< 0.0001	***
Postcrit - CritE	Fp2	-2.3960	0.4175	1891	-5.7396	< 0.0001	***
Precrit - CritE	C4	-3.8128	0.4175	1891	-9.1333	< 0.0001	***
CritB - CritE	C4	-1.9133	0.4175	1891	-4.5833	< 0.0001	***
Postcrit - CritE	C4	-3.0424	0.4175	1891	-7.2880	< 0.0001	***
Precrit - CritE	T4	-2.7878	0.4175	1891	-6.6780	< 0.0001	***
CritB - CritE	T4	-1.6697	0.4175	1891	-3.9997	0.0001955	***
Postcrit - CritE	T4	-2.2425	0.4175	1891	-5.3717	< 0.0001	***
Precrit - CritE	O2	-3.3095	0.4175	1891	-7.9277	< 0.0001	***
CritB - CritE	O2	-1.8879	0.4175	1891	-4.5223	< 0.0001	***
Postcrit - CritE	O2	-2.6731	0.4175	1891	-6.4032	< 0.0001	***
Precrit - CritE	Fp1	-4.3114	0.4175	1891	-10.3279	< 0.0001	***
CritB - CritE	Fp1	-3.2707	0.4175	1891	-7.8348	< 0.0001	***
Postcrit - CritE	Fp1	-3.4690	0.4175	1891	-8.3099	< 0.0001	***
Precrit - CritE	C3	-3.6060	0.4175	1891	-8.6380	< 0.0001	***
CritB - CritE	C3	-2.5292	0.4175	1891	-6.0586	< 0.0001	***
Postcrit - CritE	C3	-2.6094	0.4175	1891	-6.2507	< 0.0001	***
Precrit - CritE	T3	-3.0647	0.4175	1891	-7.3414	< 0.0001	***
CritB - CritE	T3	-2.2548	0.4175	1891	-5.4011	< 0.0001	***
Postcrit - CritE	T3	-1.9889	0.4175	1891	-4.7644	< 0.0001	***
Precrit - CritE	O1	-2.7948	0.4175	1891	-6.6947	< 0.0001	***
CritB - CritE	O1	-1.6341	0.4175	1891	-3.9143	0.0002782	***
Postcrit - CritE	O1	-1.6948	0.4175	1891	-4.0598	0.0001519	***

2.3.3 Linear mixed effects model diagnostics: Residual plot, Q-Q Plot, Index Plot, Histogram.

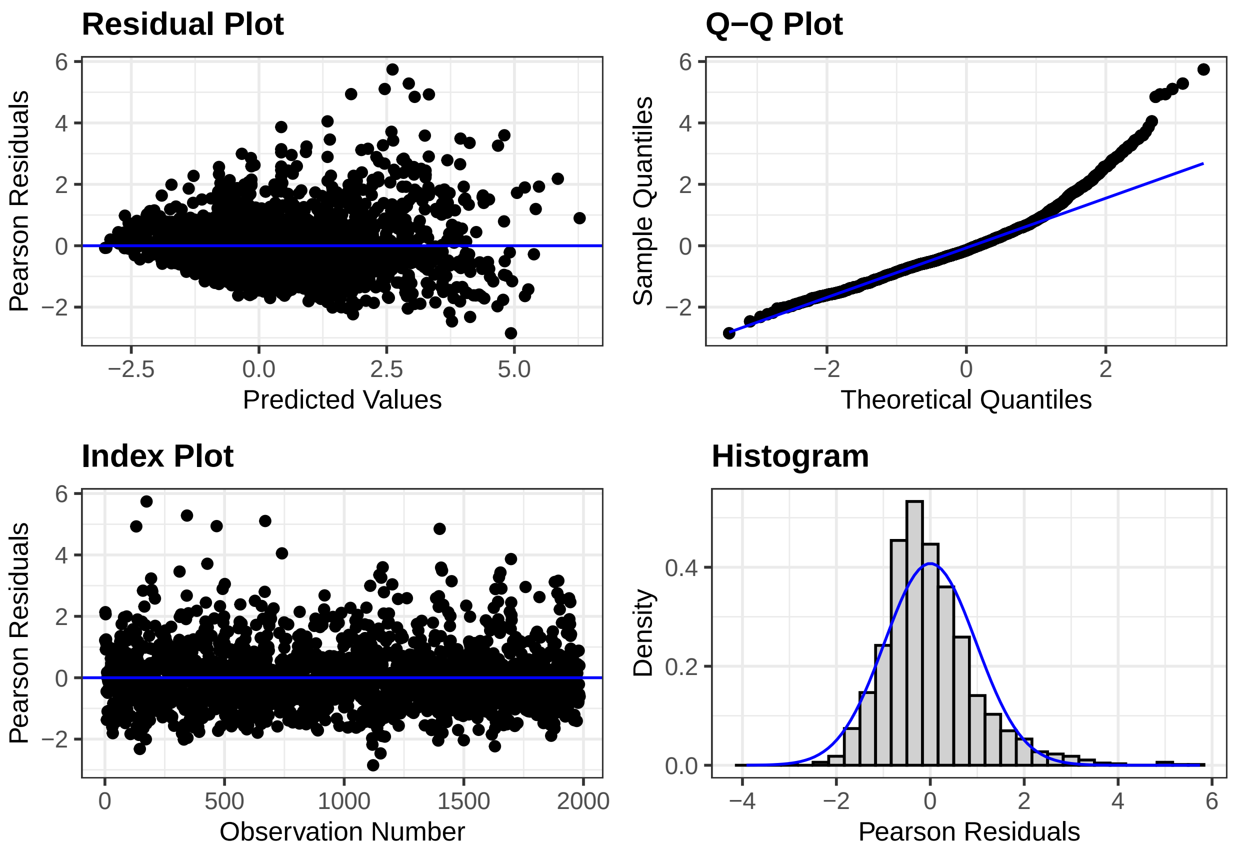

2.4 Model 4: Patient 3, no transformation

2.4.1 Type II Wald Chi-square tests

	Chisq	Df	Pr(>Chisq)	signif
States	296.6897	3	< 0.0001	***
Electrodes	59.5304	7	< 0.0001	***
States:Electrodes	137.4307	21	< 0.0001	***

2.4.2 Posthoc emmeans comparisons with Dunnett adjustment against terminal ictal phase

Contrast	Region	Estimate	SE	df	z ratio	p-value	signif
Precrit - CritE	Fp2	-0.9077	0.2884	Inf	-3.1468	0.004782	**
CritB - CritE	Fp2	-1.0564	0.2884	Inf	-3.6623	0.0007372	***
Postcrit - CritE	Fp2	-0.7137	0.2884	Inf	-2.4744	0.0369	*
Precrit - CritE	C4	-1.6635	0.2884	Inf	-5.7671	< 0.0001	***
CritB - CritE	C4	-2.0627	0.2884	Inf	-7.1512	< 0.0001	***
Postcrit - CritE	C4	-2.3127	0.2884	Inf	-8.0178	< 0.0001	***
Precrit - CritE	T4	-3.2742	0.2884	Inf	-11.3513	< 0.0001	***
CritB - CritE	T4	-3.4961	0.2884	Inf	-12.1205	< 0.0001	***
Postcrit - CritE	T4	-2.9085	0.2884	Inf	-10.0835	< 0.0001	***
Precrit - CritE	O2	0.0425	0.2884	Inf	0.1472	0.9915	ns
CritB - CritE	O2	0.1453	0.2884	Inf	0.5039	0.8938	ns
Postcrit - CritE	O2	0.2398	0.2884	Inf	0.8315	0.719	ns
Precrit - CritE	Fp1	-0.7571	0.2884	Inf	-2.6246	0.02432	*
CritB - CritE	Fp1	-1.0795	0.2884	Inf	-3.7426	0.0005381	***
Postcrit - CritE	Fp1	-0.8382	0.2884	Inf	-2.9059	0.01048	*
Precrit - CritE	C3	-1.0852	0.2884	Inf	-3.7623	0.0004977	***
CritB - CritE	C3	-1.0533	0.2884	Inf	-3.6517	0.000768	***
Postcrit - CritE	C3	-1.1957	0.2884	Inf	-4.1455	0.0001009	***
Precrit - CritE	T3	-1.4429	0.2884	Inf	-5.0023	< 0.0001	***
CritB - CritE	T3	-1.5299	0.2884	Inf	-5.3039	< 0.0001	***
Postcrit - CritE	T3	-1.4598	0.2884	Inf	-5.0609	< 0.0001	***
Precrit - CritE	O1	-1.9078	0.2884	Inf	-6.6142	< 0.0001	***
CritB - CritE	O1	-1.9652	0.2884	Inf	-6.8130	< 0.0001	***
Postcrit - CritE	O1	-2.0201	0.2884	Inf	-7.0033	< 0.0001	***

2.4.3 Linear mixed effects model diagnostics: Residual plot, Q-Q Plot, Index Plot, Histogram.

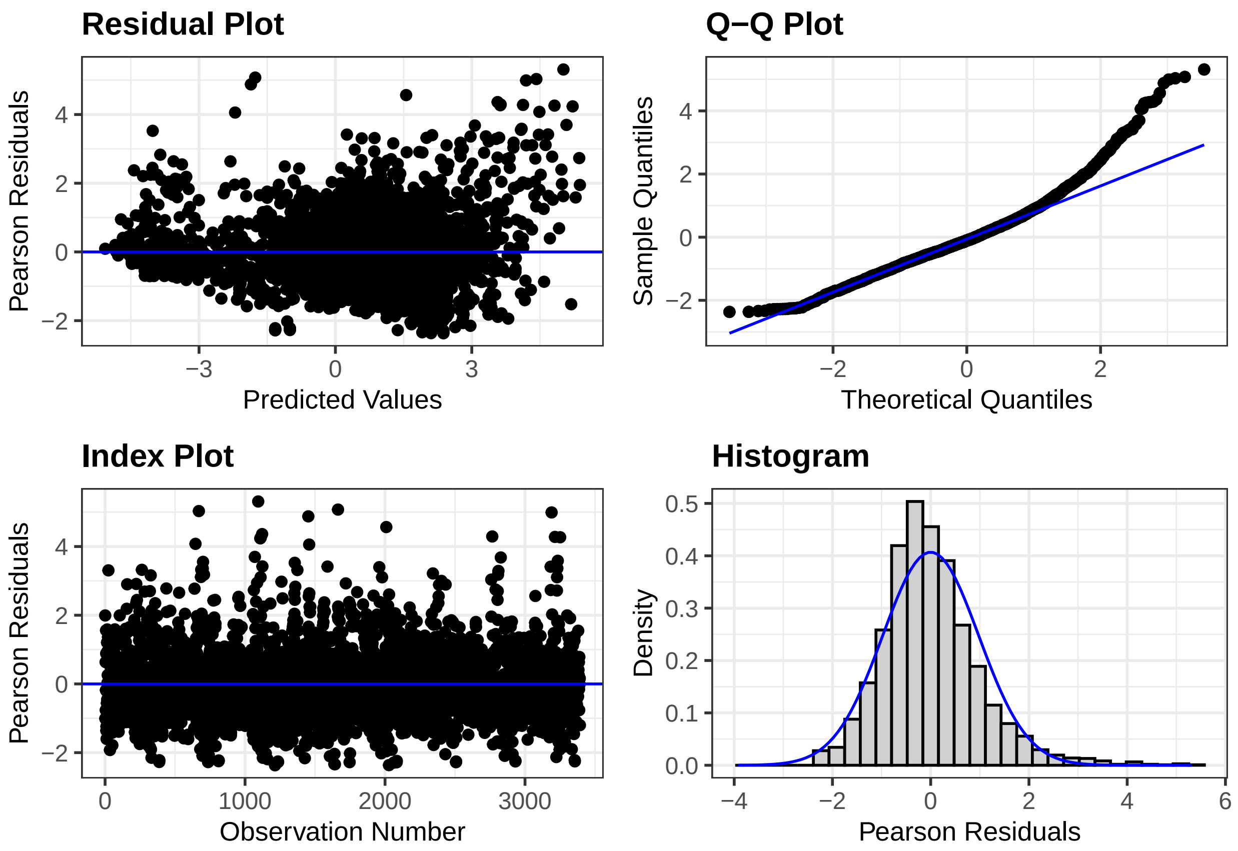

2.5 Model 5: Patient 4, no transformation

2.5.1 Type II Wald Chi-square tests

	Chisq	Df	Pr(>Chisq)	signif
States	686.6479	3	< 0.0001	***
Electrodes	57.5465	7	< 0.0001	***
States:Electrodes	72.6710	21	< 0.0001	***

2.5.2 Posthoc emmeans comparisons with Dunnett adjustment against terminal ictal phase

Contrast	Region	Estimate	SE	df	z ratio	p-value	signif
Precrit - CritE	Fp2	-1.9443	0.2666	2201	-7.2920	< 0.0001	***
CritB - CritE	Fp2	-2.6391	0.2666	2201	-9.8980	< 0.0001	***
Postcrit - CritE	Fp2	-1.5390	0.2666	2201	-5.7722	< 0.0001	***
Precrit - CritE	C4	-1.8881	0.2666	2201	-7.0813	< 0.0001	***
CritB - CritE	C4	-2.0194	0.2666	2201	-7.5736	< 0.0001	***
Postcrit - CritE	C4	-0.9144	0.2666	2201	-3.4296	0.001803	**
Precrit - CritE	T4	-3.2037	0.2666	2201	-12.0155	< 0.0001	***
CritB - CritE	T4	-3.1260	0.2666	2201	-11.7239	< 0.0001	***
Postcrit - CritE	T4	-1.6080	0.2666	2201	-6.0307	< 0.0001	***
Precrit - CritE	O2	-1.4306	0.2666	2201	-5.3655	< 0.0001	***
CritB - CritE	O2	-1.2899	0.2666	2201	-4.8379	< 0.0001	***
Postcrit - CritE	O2	0.0115	0.2666	2201	0.0430	0.9993	ns
Precrit - CritE	Fp1	-1.7908	0.2666	2201	-6.7163	< 0.0001	***
CritB - CritE	Fp1	-2.4481	0.2666	2201	-9.1816	< 0.0001	***
Postcrit - CritE	Fp1	-1.4430	0.2666	2201	-5.4120	< 0.0001	***
Precrit - CritE	C3	-2.2250	0.2666	2201	-8.3450	< 0.0001	***
CritB - CritE	C3	-2.3781	0.2666	2201	-8.9191	< 0.0001	***
Postcrit - CritE	C3	-1.0606	0.2666	2201	-3.9776	0.0002132	***
Precrit - CritE	T3	-2.0148	0.2666	2201	-7.5564	< 0.0001	***
CritB - CritE	T3	-2.0224	0.2666	2201	-7.5851	< 0.0001	***
Postcrit - CritE	T3	-0.6238	0.2666	2201	-2.3395	0.05285	ns
Precrit - CritE	O1	-1.3366	0.2666	2201	-5.0131	< 0.0001	***
CritB - CritE	O1	-1.3948	0.2666	2201	-5.2311	< 0.0001	***
Postcrit - CritE	O1	-0.0457	0.2666	2201	-0.1713	0.9885	ns

2.5.3 Linear mixed effects model diagnostics: Residual plot, Q-Q Plot, Index Plot, Histogram.

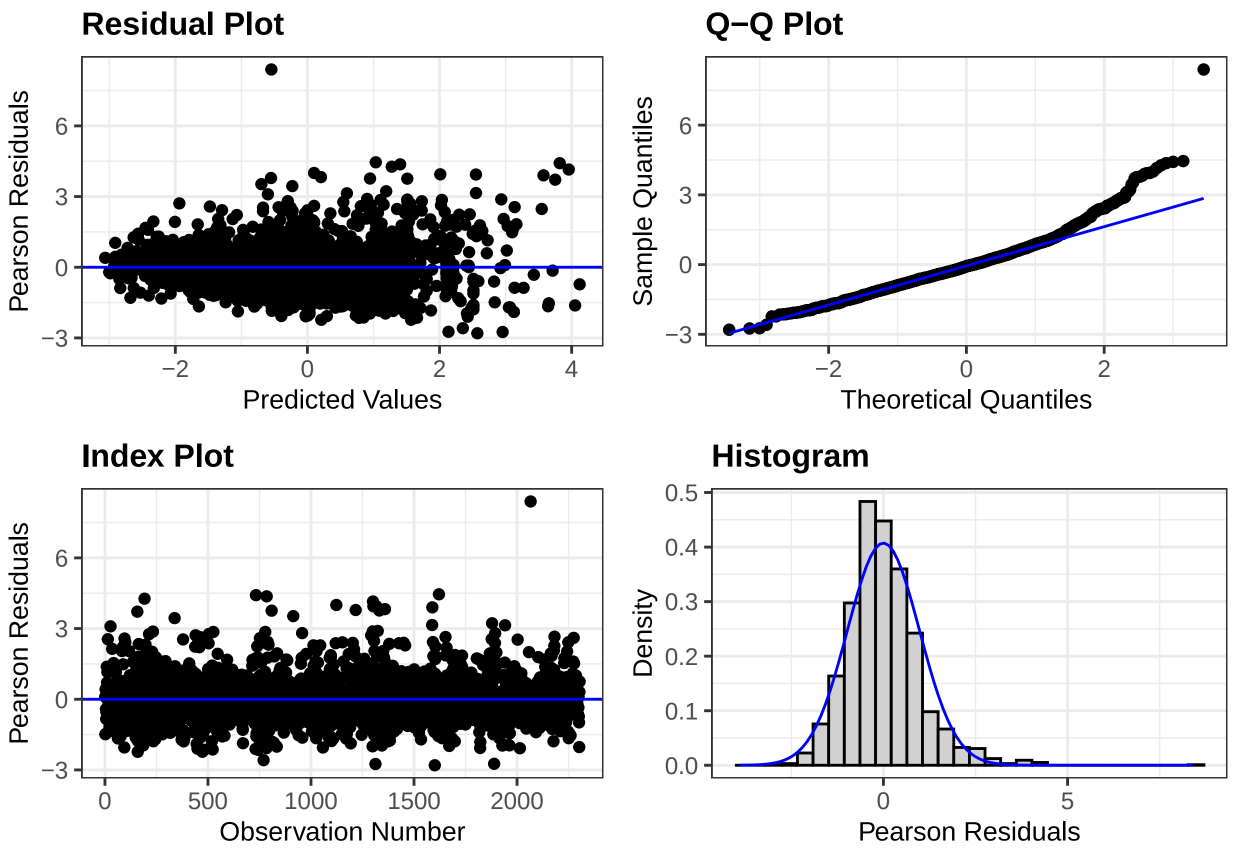

2.6 Model 6: Patient 5, no transformation

2.6.1 Type II Wald Chi-square tests

	Chisq	Df	Pr(>Chisq)	signif
States	127.0475	3	< 0.0001	***
Electrodes	19.0533	7	0.008022	**
States:Electrodes	13.8569	21	0.8757	ns

2.6.2 Posthoc emmeans comparisons with Dunnett adjustment against terminal ictal phase

Contrast	Region	Estimate	SE	df	z ratio	p-value	signif
Precrit - CritE	Fp2	-1.1626	0.281	2077	-4.1366	0.000109	***
CritB - CritE	Fp2	-1.0844	0.281	2077	-3.8586	0.000348	***
Postcrit - CritE	Fp2	-1.0045	0.281	2077	-3.5742	0.001057	**
Precrit - CritE	C4	-1.3566	0.281	2077	-4.8270	< 0.0001	***
CritB - CritE	C4	-0.8298	0.281	2077	-2.9525	0.009138	**
Postcrit - CritE	C4	-1.2752	0.281	2077	-4.5372	< 0.0001	***
Precrit - CritE	T4	-1.2002	0.281	2077	-4.2706	< 0.0001	***
CritB - CritE	T4	-0.9550	0.281	2077	-3.3981	0.002023	**
Postcrit - CritE	T4	-1.0605	0.281	2077	-3.7734	0.0004893	***
Precrit - CritE	O2	-1.0789	0.281	2077	-3.8388	0.0003769	***
CritB - CritE	O2	-0.9099	0.281	2077	-3.2377	0.003559	**
Postcrit - CritE	O2	-0.7519	0.281	2077	-2.6753	0.02117	*
Precrit - CritE	Fp1	-0.9115	0.281	2077	-3.2434	0.00349	**
CritB - CritE	Fp1	-0.9904	0.281	2077	-3.5239	0.001276	**
Postcrit - CritE	Fp1	-0.4739	0.281	2077	-1.6862	0.2242	ns
Precrit - CritE	C3	-0.6526	0.281	2077	-2.3221	0.05526	ns
CritB - CritE	C3	-0.4500	0.281	2077	-1.6013	0.2614	ns
Postcrit - CritE	C3	-0.5553	0.281	2077	-1.9757	0.125	ns
Precrit - CritE	T3	-0.8458	0.281	2077	-3.0094	0.007618	**
CritB - CritE	T3	-0.5908	0.281	2077	-2.1021	0.09416	ns
Postcrit - CritE	T3	-0.8972	0.281	2077	-3.1923	0.004157	**
Precrit - CritE	O1	-0.8968	0.281	2077	-3.1910	0.004175	**
CritB - CritE	O1	-0.7457	0.281	2077	-2.6534	0.02255	*
Postcrit - CritE	O1	-0.9069	0.281	2077	-3.2268	0.003695	**

2.6.3 Linear mixed effects model diagnostics: Residual plot, Q-Q Plot, Index Plot, Histogram.

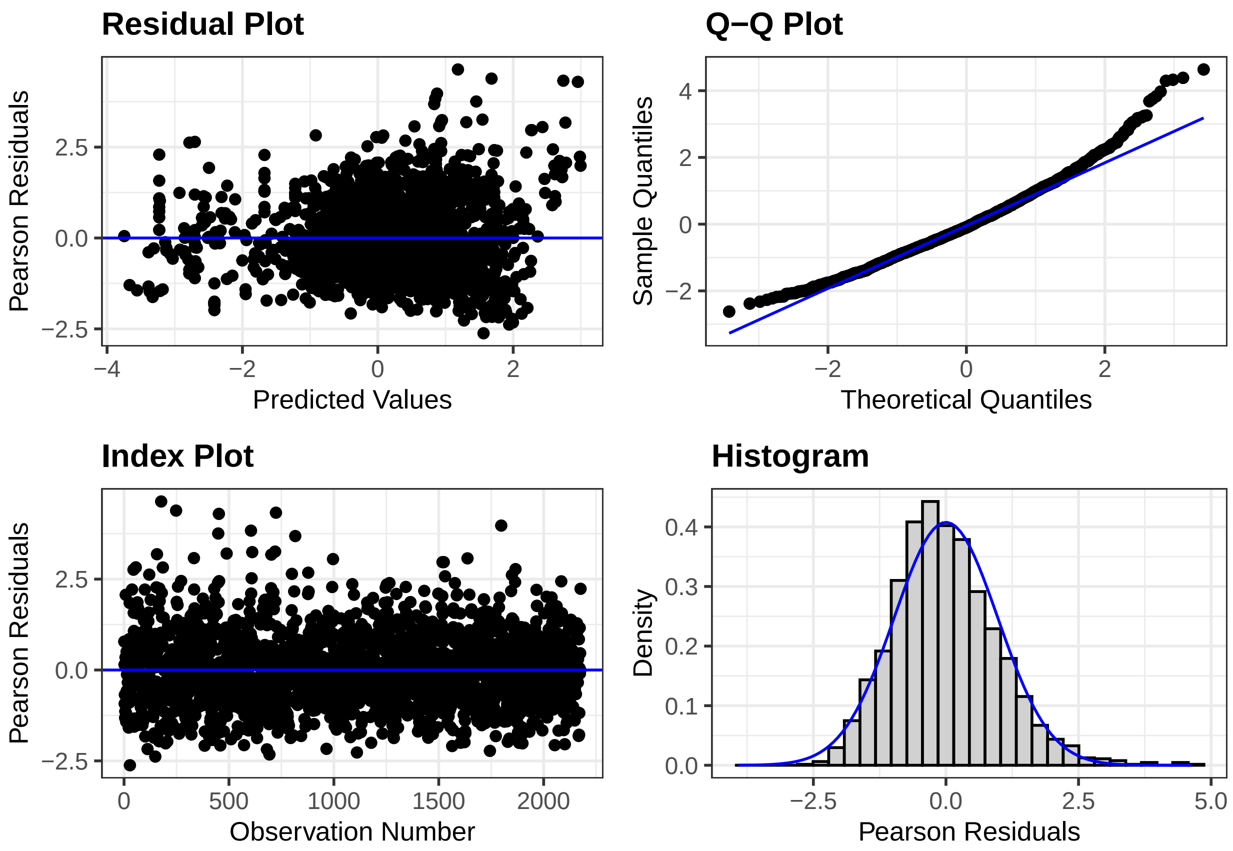

2.7 Model 7: Patient 6, no transformation

2.7.1 Type II Wald Chi-square tests

	Chisq	Df	Pr(>Chisq)	signif
States	242.0398	3	< 0.0001	***
Electrodes	57.9324	7	< 0.0001	***
States:Electrodes	135.1351	21	< 0.0001	***

2.7.2 Posthoc emmeans comparisons with Dunnett adjustment against terminal ictal phase

Contrast	Region	Estimate	SE	df	z ratio	p-value	signif
Precrit - CritE	Fp2	-2.4182	0.2655	1891	-9.1072	< 0.0001	***
CritB - CritE	Fp2	-2.3354	0.2655	1891	-8.7957	< 0.0001	***
Postcrit - CritE	Fp2	-2.9889	0.2655	1891	-11.2569	< 0.0001	***
Precrit - CritE	C4	-1.0330	0.2655	1891	-3.8903	0.0003069	***
CritB - CritE	C4	-1.0694	0.2655	1891	-4.0276	0.000174	***
Postcrit - CritE	C4	-1.4257	0.2655	1891	-5.3696	< 0.0001	***
Precrit - CritE	T4	-1.7576	0.2655	1891	-6.6194	< 0.0001	***
CritB - CritE	T4	-1.9366	0.2655	1891	-7.2936	< 0.0001	***
Postcrit - CritE	T4	-2.1771	0.2655	1891	-8.1992	< 0.0001	***
Precrit - CritE	O2	-1.3734	0.2655	1891	-5.1723	< 0.0001	***
CritB - CritE	O2	-0.9610	0.2655	1891	-3.6193	0.0008927	***
Postcrit - CritE	O2	-1.2782	0.2655	1891	-4.8139	< 0.0001	***
Precrit - CritE	Fp1	-0.2165	0.2655	1891	-0.8153	0.729	ns
CritB - CritE	Fp1	0.5836	0.2655	1891	2.1980	0.07513	ns
Postcrit - CritE	Fp1	-0.1733	0.2655	1891	-0.6528	0.8223	ns
Precrit - CritE	C3	-0.0903	0.2655	1891	-0.3401	0.9525	ns
CritB - CritE	C3	-0.2454	0.2655	1891	-0.9241	0.6609	ns
Postcrit - CritE	C3	-0.5857	0.2655	1891	-2.2060	0.07369	ns
Precrit - CritE	T3	-1.4947	0.2655	1891	-5.6293	< 0.0001	***
CritB - CritE	T3	-1.6314	0.2655	1891	-6.1442	< 0.0001	***
Postcrit - CritE	T3	-1.7257	0.2655	1891	-6.4994	< 0.0001	***
Precrit - CritE	O1	-0.3071	0.2655	1891	-1.1567	0.5109	ns
CritB - CritE	O1	-0.3256	0.2655	1891	-1.2264	0.4671	ns
Postcrit - CritE	O1	-0.5946	0.2655	1891	-2.2395	0.06792	ns

2.7.3 Linear mixed effects model diagnostics: Residual plot, Q-Q Plot, Index Plot, Histogram.

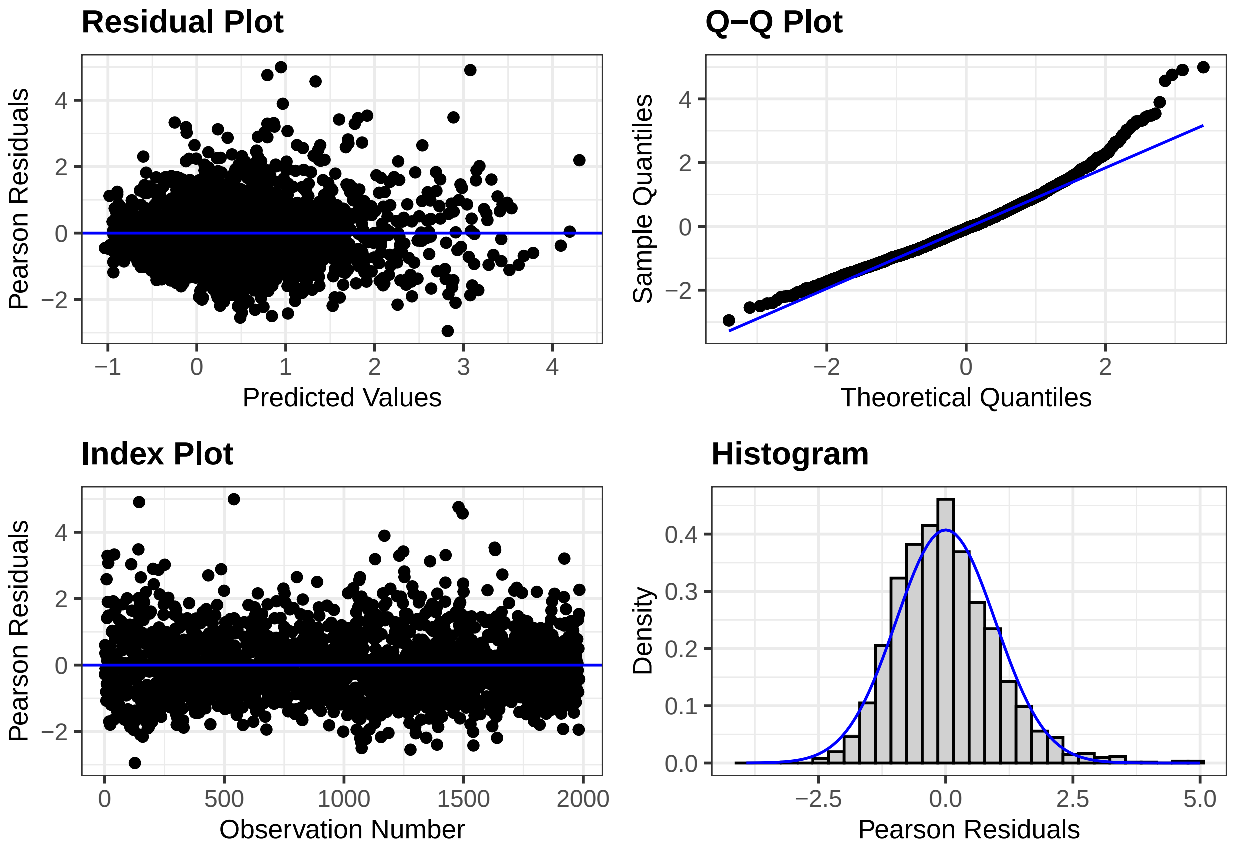

2.8 Model 8: Patient 7, no transformation

2.8.1 Type II Wald Chi-square tests

	Chisq	Df	Pr(>Chisq)	signif
States	549.5200	3	< 0.0001	***
Electrodes	67.2278	7	< 0.0001	***
States:Electrodes	176.2758	21	< 0.0001	***

2.8.2 Posthoc emmeans comparisons with Dunnett adjustment against terminal ictal phase

Contrast	Region	Estimate	SE	df	z ratio	p-value	signif
Precrit - CritE	Fp2	-1.2444	0.241	2573	-5.1632	< 0.0001	***
CritB - CritE	Fp2	-0.6470	0.241	2573	-2.6843	0.02059	*
Postcrit - CritE	Fp2	-1.3466	0.241	2573	-5.5870	< 0.0001	***
Precrit - CritE	C4	-0.7776	0.241	2573	-3.2264	0.003689	**
CritB - CritE	C4	0.4009	0.241	2573	1.6634	0.2338	ns
Postcrit - CritE	C4	-0.9916	0.241	2573	-4.1142	0.0001192	***
Precrit - CritE	T4	-1.5653	0.241	2573	-6.4946	< 0.0001	***
CritB - CritE	T4	0.0360	0.241	2573	0.1492	0.9913	ns
Postcrit - CritE	T4	-1.5980	0.241	2573	-6.6303	< 0.0001	***
Precrit - CritE	O2	-0.8546	0.241	2573	-3.5459	0.001171	**
CritB - CritE	O2	0.9391	0.241	2573	3.8965	0.0002966	***
Postcrit - CritE	O2	-1.6059	0.241	2573	-6.6631	< 0.0001	***
Precrit - CritE	Fp1	-0.1426	0.241	2573	-0.5917	0.8535	ns
CritB - CritE	Fp1	0.6994	0.241	2573	2.9018	0.0107	*
Postcrit - CritE	Fp1	-0.4944	0.241	2573	-2.0513	0.1057	ns
Precrit - CritE	C3	0.5301	0.241	2573	2.1995	0.07478	ns
CritB - CritE	C3	2.2242	0.241	2573	9.2283	< 0.0001	***
Postcrit - CritE	C3	-0.6078	0.241	2573	-2.5220	0.03257	*
Precrit - CritE	T3	-0.1124	0.241	2573	-0.4663	0.9093	ns
CritB - CritE	T3	2.0608	0.241	2573	8.5503	< 0.0001	***
Postcrit - CritE	T3	-0.4178	0.241	2573	-1.7333	0.2051	ns
Precrit - CritE	O1	0.2737	0.241	2573	1.1354	0.5244	ns
CritB - CritE	O1	1.9609	0.241	2573	8.1361	< 0.0001	***
Postcrit - CritE	O1	-0.5016	0.241	2573	-2.0814	0.0987	ns

2.8.3 Linear mixed effects model diagnostics: Residual plot, Q-Q Plot, Index Plot, Histogram.

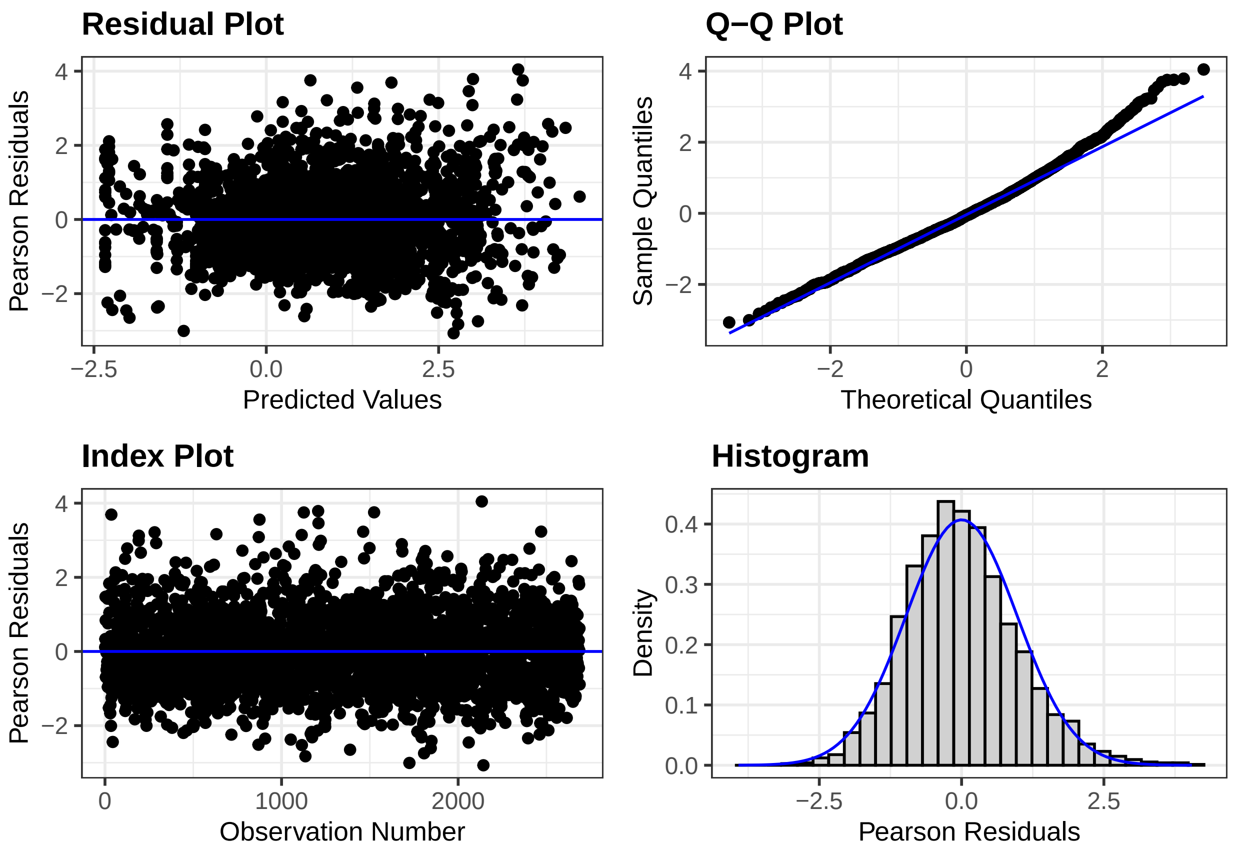

2.9 Model 9: Patient 8, no transformation

2.9.1 Type II Wald Chi-square tests

	Chisq	Df	Pr(>Chisq)	signif
States	797.6475	3	< 0.0001	***
Electrodes	460.3618	7	< 0.0001	***
States:Electrodes	411.8283	21	< 0.0001	***

2.9.2 Posthoc emmeans comparisons with Dunnett adjustment against terminal ictal phase

Contrast	Region	Estimate	SE	df	z ratio	p-value	signif
Precrit - CritE	Fp2	-0.5253	0.1034	Inf	-5.0785	< 0.0001	***
CritB - CritE	Fp2	-0.8017	0.1034	Inf	-7.7500	< 0.0001	***
Postcrit - CritE	Fp2	-0.1588	0.1034	Inf	-1.5350	0.2929	ns
Precrit - CritE	C4	-0.7980	0.1034	Inf	-7.7144	< 0.0001	***
CritB - CritE	C4	-0.9678	0.1034	Inf	-9.3561	< 0.0001	***
Postcrit - CritE	C4	-0.2179	0.1034	Inf	-2.1064	0.09296	ns
Precrit - CritE	T4	0.1539	0.1034	Inf	1.4881	0.3166	ns
CritB - CritE	T4	-0.4305	0.1034	Inf	-4.1623	< 0.0001	***
Postcrit - CritE	T4	0.6976	0.1034	Inf	6.7438	< 0.0001	***
Precrit - CritE	O2	-0.0938	0.1034	Inf	-0.9064	0.672	ns
CritB - CritE	O2	-0.7407	0.1034	Inf	-7.1609	< 0.0001	***
Postcrit - CritE	O2	0.5288	0.1034	Inf	5.1120	< 0.0001	***
Precrit - CritE	Fp1	-0.6568	0.1034	Inf	-6.3492	< 0.0001	***
CritB - CritE	Fp1	-1.1608	0.1034	Inf	-11.2222	< 0.0001	***
Postcrit - CritE	Fp1	-0.2541	0.1034	Inf	-2.4567	0.03871	*
Precrit - CritE	C3	-1.2421	0.1034	Inf	-12.0083	< 0.0001	***
CritB - CritE	C3	-1.1545	0.1034	Inf	-11.1612	< 0.0001	***
Postcrit - CritE	C3	-1.0677	0.1034	Inf	-10.3223	< 0.0001	***
Precrit - CritE	T3	-1.2413	0.1034	Inf	-12.0007	< 0.0001	***
CritB - CritE	T3	-1.3094	0.1034	Inf	-12.6583	< 0.0001	***
Postcrit - CritE	T3	-0.7398	0.1034	Inf	-7.1522	< 0.0001	***
Precrit - CritE	O1	0.3975	0.1034	Inf	3.8429	0.0003601	***
CritB - CritE	O1	-0.6081	0.1034	Inf	-5.8789	< 0.0001	***
Postcrit - CritE	O1	0.8105	0.1034	Inf	7.8354	< 0.0001	***

2.9.3 Linear mixed effects model diagnostics: Residual plot, Q-Q Plot, Index Plot, Histogram.

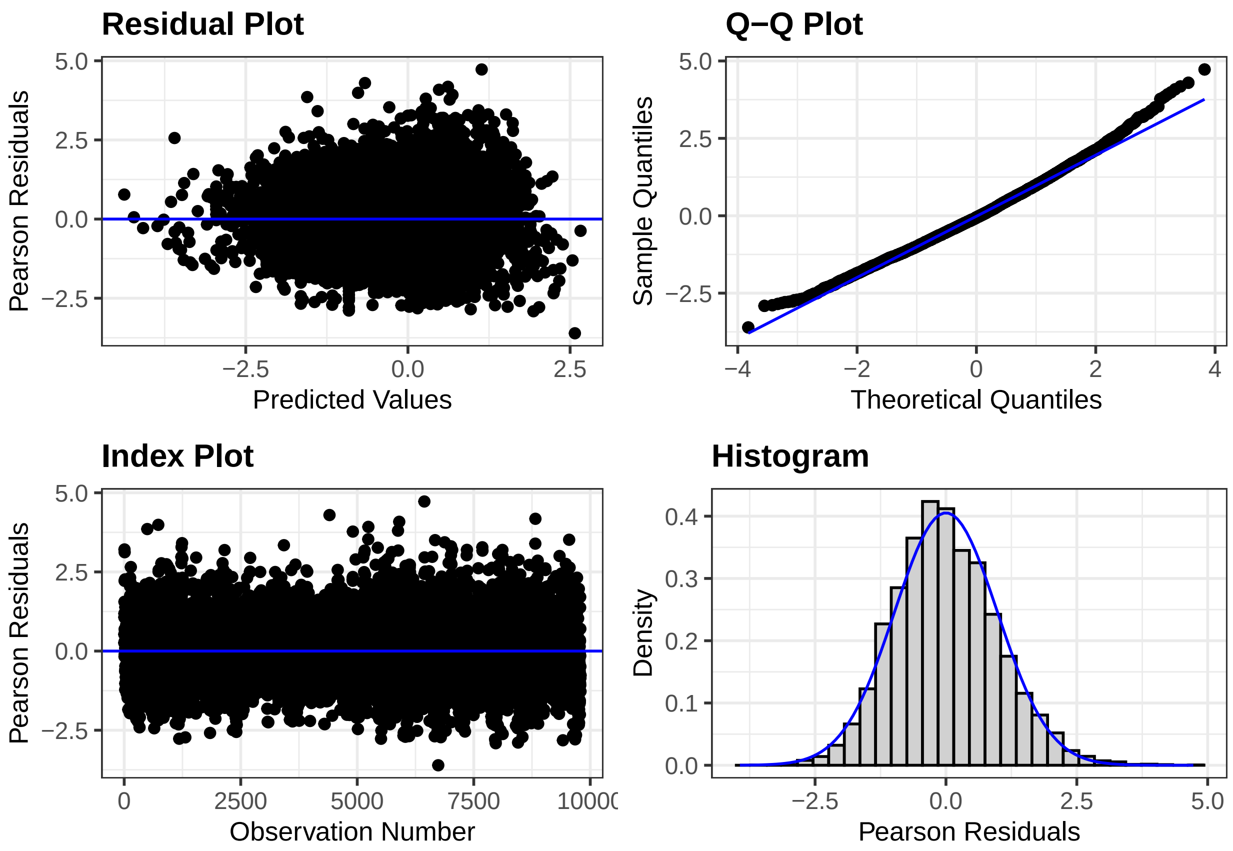

2.10 Model 10: Patient 9, no transformation

2.10.1 Type II Wald Chi-square tests

	Chisq	Df	Pr(>Chisq)	signif
States	21.3847	3	< 0.0001	***
Electrodes	8.1753	7	0.3174	ns
States:Electrodes	10.5151	21	0.9714	ns

2.10.2 Posthoc emmeans comparisons with Dunnett adjustment against terminal ictal phase

Contrast	Region	Estimate	SE	df	z ratio	p-value	signif
Precrit - CritE	Fp2	-0.0965	0.1867	Inf	-0.5166	0.8883	ns
CritB - CritE	Fp2	0.0378	0.1867	Inf	0.2025	0.9837	ns
Postcrit - CritE	Fp2	-0.3778	0.1867	Inf	-2.0234	0.1123	ns
Precrit - CritE	C4	0.3008	0.1867	Inf	1.6112	0.2566	ns
CritB - CritE	C4	0.1766	0.1867	Inf	0.9456	0.6469	ns
Postcrit - CritE	C4	-0.1462	0.1867	Inf	-0.7830	0.7484	ns
Precrit - CritE	T4	-0.0322	0.1867	Inf	-0.1724	0.9883	ns
CritB - CritE	T4	-0.0115	0.1867	Inf	-0.0616	0.9986	ns
Postcrit - CritE	T4	-0.2528	0.1867	Inf	-1.3537	0.3904	ns
Precrit - CritE	O2	-0.0193	0.1867	Inf	-0.1035	0.9959	ns
CritB - CritE	O2	0.0979	0.1867	Inf	0.5242	0.885	ns
Postcrit - CritE	O2	-0.1610	0.1867	Inf	-0.8622	0.6999	ns
Precrit - CritE	Fp1	0.2856	0.1867	Inf	1.5298	0.2954	ns
CritB - CritE	Fp1	0.0409	0.1867	Inf	0.2191	0.9809	ns
Postcrit - CritE	Fp1	0.0488	0.1867	Inf	0.2614	0.9724	ns
Precrit - CritE	C3	-0.0540	0.1867	Inf	-0.2893	0.966	ns
CritB - CritE	C3	0.1292	0.1867	Inf	0.6919	0.801	ns
Postcrit - CritE	C3	-0.2897	0.1867	Inf	-1.5518	0.2846	ns
Precrit - CritE	T3	-0.1497	0.1867	Inf	-0.8018	0.7371	ns
CritB - CritE	T3	-0.1353	0.1867	Inf	-0.7249	0.7824	ns
Postcrit - CritE	T3	-0.3901	0.1867	Inf	-2.0896	0.09665	ns
Precrit - CritE	O1	0.0515	0.1867	Inf	0.2760	0.9692	ns
CritB - CritE	O1	0.1042	0.1867	Inf	0.5581	0.8696	ns
Postcrit - CritE	O1	-0.1496	0.1867	Inf	-0.8013	0.7374	ns

2.10.3 Linear mixed effects model diagnostics: Residual plot, Q-Q Plot, Index Plot, Histogram.

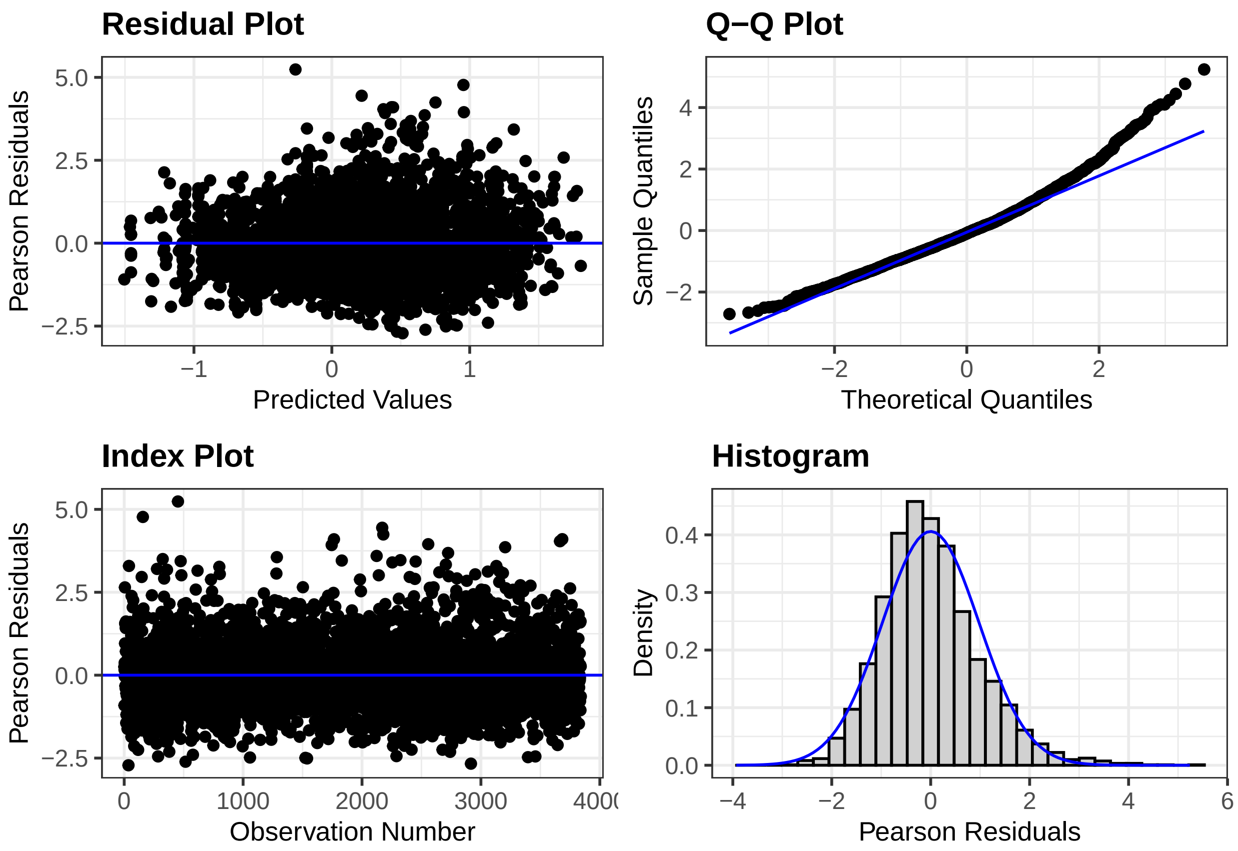

2.11 Model 11: Patient 10, no transformation

2.11.1 Type II Wald Chi-square tests

	Chisq	Df	Pr(>Chisq)	signif
States	5.3967	3	0.145	ns
Electrodes	17.3773	7	0.01512	*
States:Electrodes	25.2510	21	0.2365	ns

2.11.2 Posthoc emmeans comparisons with Dunnett adjustment against terminal ictal phase

Contrast	Region	Estimate	SE	df	z ratio	p-value	signif
Precrit - CritE	Fp2	-0.0413	0.1944	2821	-0.2126	0.982	ns
CritB - CritE	Fp2	-0.2769	0.1944	2821	-1.4241	0.3509	ns
Postcrit - CritE	Fp2	-0.1886	0.1944	2821	-0.9699	0.6314	ns
Precrit - CritE	C4	-0.3563	0.1944	2821	-1.8324	0.1688	ns
CritB - CritE	C4	-0.2305	0.1944	2821	-1.1856	0.4925	ns
Postcrit - CritE	C4	-0.0181	0.1944	2821	-0.0932	0.9967	ns
Precrit - CritE	T4	-0.2538	0.1944	2821	-1.3054	0.4189	ns
CritB - CritE	T4	-0.3525	0.1944	2821	-1.8126	0.1756	ns
Postcrit - CritE	T4	-0.0137	0.1944	2821	-0.0706	0.9981	ns
Precrit - CritE	O2	-0.1781	0.1944	2821	-0.9159	0.6661	ns
CritB - CritE	O2	-0.3778	0.1944	2821	-1.9427	0.1341	ns
Postcrit - CritE	O2	-0.0713	0.1944	2821	-0.3666	0.9446	ns
Precrit - CritE	Fp1	0.2409	0.1944	2821	1.2389	0.4592	ns
CritB - CritE	Fp1	-0.0255	0.1944	2821	-0.1313	0.9933	ns
Postcrit - CritE	Fp1	-0.0952	0.1944	2821	-0.4896	0.8999	ns
Precrit - CritE	C3	0.4750	0.1944	2821	2.4428	0.04032	*
CritB - CritE	C3	0.2517	0.1944	2821	1.2946	0.4254	ns
Postcrit - CritE	C3	0.1429	0.1944	2821	0.7347	0.7769	ns
Precrit - CritE	T3	0.2090	0.1944	2821	1.0747	0.5635	ns
CritB - CritE	T3	0.1556	0.1944	2821	0.8002	0.7381	ns
Postcrit - CritE	T3	-0.0155	0.1944	2821	-0.0799	0.9976	ns
Precrit - CritE	O1	-0.1784	0.1944	2821	-0.9176	0.665	ns
CritB - CritE	O1	-0.2886	0.1944	2821	-1.4843	0.3188	ns
Postcrit - CritE	O1	-0.1013	0.1944	2821	-0.5208	0.8865	ns

2.11.3 Linear mixed effects model diagnostics: Residual plot, Q-Q Plot, Index Plot, Histogram.

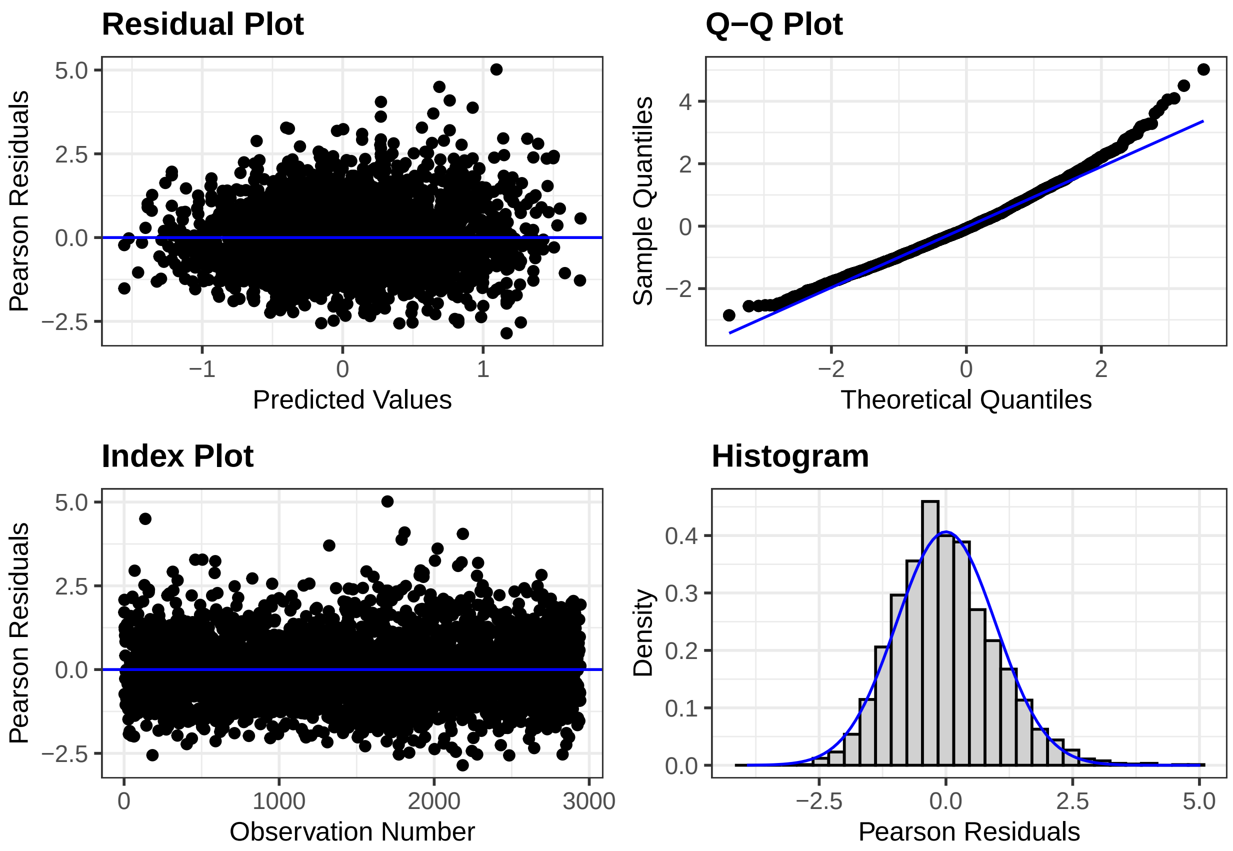

Supplementary

Imaginary coherence calculation

From Nolte et al., Clinical Neurophysiology 2004

Coherency between two EEG-channels is a measure of the linear relationship of the two at a specific frequency.

First let us define the complex Fourier transform of a time serie $\hat{x}_i(t)$ estimated on a time epoch between t_1 and t_2 as:

$$x_i(f) = \int_{t_1}^{t_2} \hat{x}_i(t)e^{-i2\pi ft}\, dt$$

Let $x_i(f)$ and $\chi_j(f)$ be the complex Fourier transforms of the time series $\hat{x}_i(t)$ and $\hat{x}_j(t)$ of channel i and j, respectively. * represents the complex conjugate of a complex number and $\langle \quad \rangle$ the expected value i.e. the mean over time/epochs. Then the cross-spectrum of the two time series is defined as:

$$S_{ij}(f) \equiv \langle x_i(f)\chi_j^*(f)\rangle$$

Coherency is now defined as the normalized cross-spectrum

$$C_{ij}(f) \equiv \frac{S_{ij}(f)}{\left(S_{ii}(f)S_{jj}(f)\right)^{1/2}}$$

and coherence is defined as the absolute value of coherency.

$$Coh_{ij}(f) = \left|C_{jj}(f)\right|$$

Imaginary coherence is then defined as the imaginary part of coherency:

$$iCoh_{ij}(f) = Im\left[C_{ij}(f)\right]$$

For a given frequency band ranging from f_1 to f_2 , the Imaginary coherence over the frequency band is defined as the mean of the imaginary coherence over all the frequencies of the band.
